# Public Water Arsenic and Birth Outcomes in the Environmental Influences on Child Health Outcomes Cohort

**DOI:** 10.1001/jamanetworkopen.2025.14084

**Published:** 2025-06-16

**Authors:** Anne E. Nigra, Tessa R. Bloomquist, Tushara Rajeev, Mohamad Burjak, Joan A. Casey, Dana E. Goin, Julie B. Herbstman, Yoshira Ornelas Van Horne, Blair J. Wylie, Ilan Cerna-Turoff, Joseph M. Braun, Kristen L. McArthur, Margaret R. Karagas, Jennifer L. Ames, Allison R. Sherris, Catherine M. Bulka, Amy M. Padula, Caitlin G. Howe, Rebecca C. Fry, Lauren A. Eaves, Carrie V. Breton, Andrea E. Cassidy-Bushrow, Johnnye Lewis, Debra MacKenzie, Daniel Beene, Shohreh F. Farzan, Sheela Sathyanarayana, Alison E. Hipwell, Rachel Morello-Frosch, Brittney M. Snyder, Tina V. Hartert, Amy J. Elliott, Thomas G. O’Connor, Amii M. Kress

**Affiliations:** 1 Department of Environmental Health Sciences, Columbia University Mailman School of Public Health, New York, New York; 2 Department of Epidemiology, Johns Hopkins Bloomberg School of Public Health, Baltimore, Maryland; 3 Department of Environmental and Occupational Health Sciences, University of Washington School of Public Health, Seattle; 4 Department of Epidemiology, Columbia University Mailman School of Public Health, New York, New York; 5 Department of Epidemiology, Brown University, Providence, Rhode Island; 6 Department of Epidemiology, Geisel School of Medicine at Dartmouth College, Lebanon, New Hampshire; 7 Division of Research, Kaiser Permanente Northern California, Pleasanton; 8 College of Public Health, University of South Florida, Tampa; 9 Program for Reproductive Health and the Environment, Department of Obstetrics, Gynecology and Reproductive Sciences, University of California, San Francisco; 10 Department of Environmental Sciences and Engineering, University of North Carolina at Chapel Hill; 11 Department of Population and Public Health Sciences, Keck School of Medicine of University of Southern California, Los Angeles; 12 Department of Public Health Sciences, Henry Ford Health, Detroit, Michigan; 13 Department of Pediatrics and Human Development, College of Human Medicine, Michigan State University, East Lansing; 14 Community Environmental Health Program, College of Pharmacy, University of New Mexico Health Sciences Center, Albuquerque; 15 Department of Pediatrics, Environmental and Occupational Health Sciences and Seattle Children’s Research Institute, Seattle, Washington; 16 Department of Psychiatry, University of Pittsburgh, Pittsburgh, Pennsylvania; 17 Department of Psychology, University of Pittsburgh, Pittsburgh, Pennsylvania; 18 Department of Clinical and Translation Science, University of Pittsburgh, Pittsburgh, Pennsylvania; 19 Department of Environmental Science, Policy and Management, University of California, Berkeley; 20 School of Public Health, University of California, Berkeley; 21 Department of Medicine, Vanderbilt University Medical Center, Nashville, Tennessee; 22 Department of Pediatrics, Vanderbilt University Medical Center, Nashville, Tennessee; 23 Avera Research Institute, Sioux Falls, South Dakota; 24 Department of Pediatrics, University of South Dakota Sanford School of Medicine, Sioux Falls; 25 Department of Psychiatry, University of Rochester, Rochester, New York; 26 Department of Neuroscience, University of Rochester, Rochester, New York; 27 Department of Obstetrics and Gynecology, University of Rochester, Rochester, New York

## Abstract

**Importance:**

Inorganic arsenic is associated with adverse birth outcomes, but evidence is limited for public water concentrations (modifiable by federal regulatory action) in US populations.

**Objective:**

To evaluate the association between prenatal public water arsenic exposure below the federal regulatory standard of 10 μg/L and birth outcomes in the US.

**Design, Setting, and Participants:**

This cohort study analyzed observational pregnancy cohort data from the Environmental Influences on Child Health Outcomes (ECHO) Cohort for birthing parent–infant dyads from 35 pregnancy cohort sites. Infants were born between 2005 and 2020. The data were analyzed between 2024 and 2025.

**Exposure:**

Individual, time-weighted, mean prenatal public water arsenic exposures were estimated by joining Zip Code Tabulation Area–level public water arsenic concentrations with monthly residential history data during pregnancy.

**Main Outcome and Measure:**

Adjusted risk ratios (RRs) of preterm birth, low birth weight, and small for gestational age were evaluated. Adjusted RRs, mean differences in birth weight–for–gestational age *z* score and birth weight, and the geometric mean ratio of gestational age at birth were calculated via cubic splines, per 1 μg/L higher prenatal water arsenic, and across policy-relevant categories of exposure.

**Results:**

The cohort comprised 13 998 birthing parents (mean [SD] age, 30.8 [5.6] years) of whom 4.5% were of American Indian, Alaska Native, Native Hawaiian, or Pacific Islander; 7.2% Asian; 12.4% Black; 56.1% White; 4.2% multiple races; and 8.5% another race and 28.1% were of Hispanic/Latino and 70.4% non-Hispanic/Latino ethnicity. Prenatal public water arsenic ranged from less than 0.35 to 37.28 μg/L. In spline models, prenatal public water arsenic was associated with a higher risk of low birth weight, lower birth weight, and lower birth weight–for–gestational age *z* score, although effect estimates lacked precision. The RR of low birth weight per 1 μg/L higher prenatal water arsenic was higher among Black (1.02; 95% CI, 1.01-1.03), Hispanic/Latino (1.07; 95% CI 1.02-l.12), and White (1.04; 95% CI, 102-1.06) birthing parents, and the RR for preterm birth was higher among Hispanic/Latino birthing parents (1.05; 95% CI, 1.01-1.09). The mean difference of birth weight and birth weight–for–gestational age *z* score per 1 μg/L higher prenatal water arsenic was more pronounced among White birthing parents (−10 g [95% CI, −17 to −3 g]; −0.02 SDs [95% CI −0.03 to −0.01 SDs]). No evidence that prenatal public water arsenic mediated the association between birthing parent race and ethnicity and adverse birth outcomes was observed.

**Conclusions and Relevance:**

In this cohort study of birthing parent–infant dyads across the US, arsenic measured in public water systems was associated with birth outcomes at levels below the current US Environmental Protection Agency’s maximum contaminant level. The findings suggest that further reducing the maximum contaminant level for arsenic may decrease the number of infants with low birth weight in the US.

## Key Points

**Question:**

Is prenatal exposure to arsenic in public drinking water associated with birth outcomes in the US?

**Findings:**

In this cohort study of 13 998 birthing parent–infant dyads from the Environmental Influences on Child Health Outcomes Cohort, higher prenatal public water arsenic levels were associated with lower mean birth weight and birth weight–for–gestational age *z* score and a higher risk of low birth weight. Associations were modified by race and ethnicity.

**Meaning:**

These findings suggest that public water arsenic exposure is associated with adverse birth outcomes, even at levels below the current US Environmental Protection Agency maximum contaminant level.

## Teaser

This cohort study evaluates the association between prenatal exposure to public water arsenic below federal regulatory standards and birth outcomes in the Environmental Influences on Child Health Outcomes (ECHO) Cohort.

## Introduction

Regulated US public drinking water is a major source of exposure to inorganic arsenic.^1,2^ Prenatal water arsenic exposure is associated with adverse birth outcomes at moderate to high concentrations (>50 μg/L), including preterm birth and low birth weight,^3,4,5^ which are important predictors of infant mortality and morbidity across the lifespan. These associations persist at lower exposure levels common in US populations (<10 μg/L), although evidence is more limited.^5^ Available US evidence has been limited to private well exposures (which are not federally regulated)^6,7^; studies that used urinary biomarkers of exposure (which may be biased because arsenic influences kidney function and excretion, and which integrate all exposure sources, making regulatory insights challenging)^8^; studies of drinking water violations rather than continuous exposures^9^; and single-site studies with limited racial, ethnic, and geographic diversity.^10^

The US Environmental Protection Agency (EPA) considers cost, technical feasibility, and public health benefit when setting maximum contaminant levels (MCLs) for public water systems.^11^ The EPA MCL for arsenic is 10 μg/L, although the MCL goal (determined only by public health benefit) is 0 μg/L (reflecting that there is no safe level of exposure^5^). New Jersey, New Hampshire, and Denmark set MCLs of 5 μg/L, and the MCL for the Netherlands is 1 μg/L.^2^ Birth outcomes are considered quantitatively in the EPA’s draft inorganic arsenic risk assessment (1 study was considered suitable for dose-response analysis).^5^ A substantial gap in the current literature is an evaluation of the association between public water arsenic concentrations (as measured by water systems to comply with the MCL) and birth outcomes in diverse US populations with arsenic levels below the current MCL.^5^

Our objective was to evaluate the association between estimated prenatal public drinking water arsenic concentrations and birth outcomes in the Environmental Influences on Child Health Outcomes (ECHO) Cohort.^12^ We evaluated the association of individual-level, time-weighted, mean prenatal public water arsenic with preterm birth, low birth weight, small for gestational age (SGA), and birth weight for gestational age (primary outcomes) and gestational age at birth and birth weight (secondary outcomes). To evaluate potential heterogeneity in the association across subgroups, we stratified our analysis by birthing parent race, ethnicity, educational attainment, and area-level socioeconomic vulnerability. We additionally assessed racial and ethnic disparities in birth outcomes within the ECHO Cohort and explored whether prenatal public water arsenic might mediate persistent racial and ethnic disparities in these outcomes.

## Methods

### ECHO Cohort

This cohort study involved the ECHO Cohort (cycle 1), which comprises 69 ongoing pregnancy and pediatric cohort sites across the US and Puerto Rico.^12^ Study protocols were approved by local or the ECHO institutional review board, and written informed parental consent or permission was obtained. This study was approved by the Columbia University Institutional Review Board and followed the Strengthening the Reporting of Observational Studies in Epidemiology (STROBE) reporting guideline for cohort studies.

Birthing parents and infants were eligible for our study if parents were recruited to a general population cohort site, had a reported residential address during pregnancy, and resided in a Zip Code Tabulation Area (ZCTA) with public water arsenic exposure data available.^13^ We excluded infants missing outcome data, nonsingleton pregnancies, births before 2005 (to align exposure data), births with residential histories covering less than 80% of the pregnancy period, births from parents who moved across state lines during pregnancy, births from cohorts with fewer than 25 eligible participants, and births from 1 cohort reliant almost exclusively on private wells. The final sample size was 13 998 births from 35 cohort sites (eFigure 1 in Supplement 1).

### Exposure Assessment: Prenatal Public Water Arsenic

We leveraged previously developed ZCTA-level estimates of mean, population-weighted public water arsenic concentrations,^13^ which we developed from routine compliance monitoring records collected by public water systems to ensure compliance with the MCL and reported to the EPA.^13^ These estimates were previously validated in 2 multisite cohorts with urinary biomarkers reflecting internal dose.^1,13^ To reduce differential missingness and bias,^14^ ZCTA-level estimates were aggregated to 3-year periods corresponding to the EPA Standardized Monitoring Framework from 2006 through 2019 (eg, 2014-2016, 2017-2019).^13,15^ Zip Code Tabulation Area–level estimates less than or equal to 0.35 μg/L reflect either (1) arsenic concentrations measured below the limit of detection or (2) very low arsenic concentrations measured above the detection limit at laboratories with unusually high precision and low detection limits.

To create individual, time-weighted, mean prenatal public water arsenic exposure estimates, we assigned ZCTA-level public water arsenic estimates to each prenatal month using geocoded birthing parent residential address. This approach accounted for residential moves across ZCTAs and time-varying exposures for pregnancies spanning multiple exposure periods. We could not assign ZCTA-level public water arsenic estimates to birthing parents in 1 cohort because participants often relied on hauling water from regulated sources across ZCTA boundaries.^16^ We assigned these participants to public water system–level estimates^14^ according to self-reported drinking water source (which was previously matched to public water systems)^16^ and excluded these participants in sensitivity analyses. Detailed exposure assignment information was previously published^13^ and is summarized in the eMethods in Supplement 1.

### Birth Outcomes

The primary outcomes were preterm birth (gestational age <37 weeks), low birth weight (<2500 g, clinically relevant but confounded by gestational age), SGA (sex-specific birth weight for gestational age <10th percentile),^17^ and birth weight–for–gestational age *z* score (sex-specific, continuous).^17^ We additionally evaluated birth weight (continuous, in grams) and gestational age at birth (continuous, in weeks) as complementary analyses to our primary analyses with clinically relevant outcomes (preterm birth, low birth weight, SGA). Outcome data were collected by each cohort site based on its protocols and were derived from several sources, including medical records and birthing parent or caregiver self-report.^12^

### Covariates

Individual-level covariates included infant sex, conception season, birthing parent education (high school diploma or less or equivalent, some college or an associate’s degree, or bachelor’s degree or higher), age at delivery (<25 years, 25-29 years, 30-34 years, ≥35 years), prepregnancy body mass index, prenatal tobacco use (any, none), parity (nulliparous, multiparous), self-reported birth parent race (combined American Indian, Alaska Native, Native Hawaiian or Pacific Islander due to small sample sizes; Asian; Black; White; multiple races; and another race) and ethnicity (Hispanic/Latino, non-Hispanic/Latino), and prenatal public water uranium estimates (continuous, micrograms per liter) (eMethods in Supplement 1). Area-level covariates included ZCTA-level population density (population divided by land area in square miles) and census tract socioeconomic vulnerability domain score (continuous). The socioeconomic vulnerability domain score was developed by the Centers for Disease Control and Prevention/Agency for Toxic Substances and Disease Registry, which derived scores from US Census American Community Survey estimates of median household income and the percentage of adults who are unemployed, are living below the poverty line, and do not have a high school diploma.^18^

### Statistical Analysis

All data management and analyses were conducted using R, version 4.4.0 (R Foundation for Statistical Computing) between 2024 and 2025. We evaluated associations between prenatal public water arsenic and birth outcomes with linear (continuous outcomes) and Poisson (binary outcomes, robust variance)^19,20^ generalized estimating equation models with exchangeable correlation structure to account for clustering of birthing parents by cohort site. We evaluated the adjusted risk ratio (RR) of preterm birth, low birth weight, and SGA, the mean difference in birth weight–for–gestational age *z* score and birth weight (normally distributed), and the percent change in the geometric mean ratio of gestational age (natural logarithm normally distributed). Our primary models evaluated prenatal public water arsenic continuously, using cubic splines to allow for a nonlinear exposure-response association. We set the reference to 0.35 μg/L (corresponding to undetectable public water arsenic concentrations) and included knots at the 67th and 83rd percentiles of the prenatal water arsenic distribution (2 equally spaced knots between the reference and maximum). We also evaluated associations of continuous linear (per 1.00 and 5.00 μg/L higher prenatal public water arsenic) and categorical (>0.35 to 1.00 μg/L, >1.00 to 2.00 μg/L, >2.00 to 5.00 μg/L, and >5.00 μg/L vs reference group of ≤0.35 μg/L) arsenic concentrations with birth outcomes. In progressively adjusted models, model 1 adjusted for birthing parent age and education and infant sex. Model 2 further adjusted for parity, prepregnancy body mass index, prenatal tobacco use, season of conception, and ZCTA-level population density. As race and ethnicity are social constructs, we did not adjust for birthing parent race or ethnicity because this approach may obscure racial and ethnic disparities in both the exposure and outcome and, in effect, perpetuate them.^21^ For all models, we used multiple imputation by chained equations (5 iterations and 25 imputed datasets) to impute missing covariates. In interpreting effect estimates, we followed best current practice and evaluated the direction, magnitude, consistency, and precision of effect estimates instead of relying on dichotomous significance testing.^22^

#### Subgroup Analyses

For models evaluating associations per 1.00 μg/L higher prenatal public water arsenic, we further stratified by birthing parent race, ethnicity, education, and area-level socioeconomic vulnerability index to evaluate potential differential effects of prenatal public water arsenic on adverse birth outcomes across subgroups (effect measure modification). We used model 2 adjustments (birthing parent education, age, parity, prepregnancy birthing parent body mass index, prenatal tobacco use, season of birth, and ZCTA population density). Differential effects would be related to other social and environmental exposures inequitably distributed across racial, ethnic, and socioeconomic subgroups^21^ (eg, chemical exposures,^23^ racism and discrimination^24^). Subgroup analyses were limited by a small number of cases and were therefore unable to evaluate flexible spline models. We did not report effect estimates for subgroups with fewer than 50 participants.

We also explored whether prenatal public water arsenic exposure mediated persistent and well-established racial and ethnic disparities in adverse birth outcomes.^25^ We first quantified racial and ethnic disparities within our analytic sample by evaluating effect estimates for each outcome comparing birthing parent race, ethnicity, and combined race and ethnicity with a reference group that benefits from white supremacy (specifically, White and non-Hispanic/Latino birthing parents), using model 2 adjustments.^26^ We used the GEEmediate package in R to estimate natural direct and indirect effects.

#### Sensitivity Analyses

We additionally adjusted for prenatal public drinking water uranium exposure (often correlated with arsenic), and considered alternative knot points for spline models (60th and 90th percentiles). We also restricted our analyses to birthing parents (1) with the highest geocoding quality; (2) who resided in states that publish shapefiles of public water system distribution boundaries, for whom measurement error of the exposure is smaller (eMethods in Supplement 1)^13^; (3) who did not move during pregnancy; (4) who did not move during pregnancy and who resided in states that publish shapefiles of public water system distribution boundaries; and (5) who reported that their tap water source was a public system (a subset of 1025 of 1193 with data on tap water source available). We also report results from sensitivity analyses removing each cohort site (leave-one-out analysis) to identify influential cohorts, conducting g-computation for intervention effects, and repeating our analyses using census tract–level exposure data.

## Results

### Participant Characteristics

**Figure 1.**
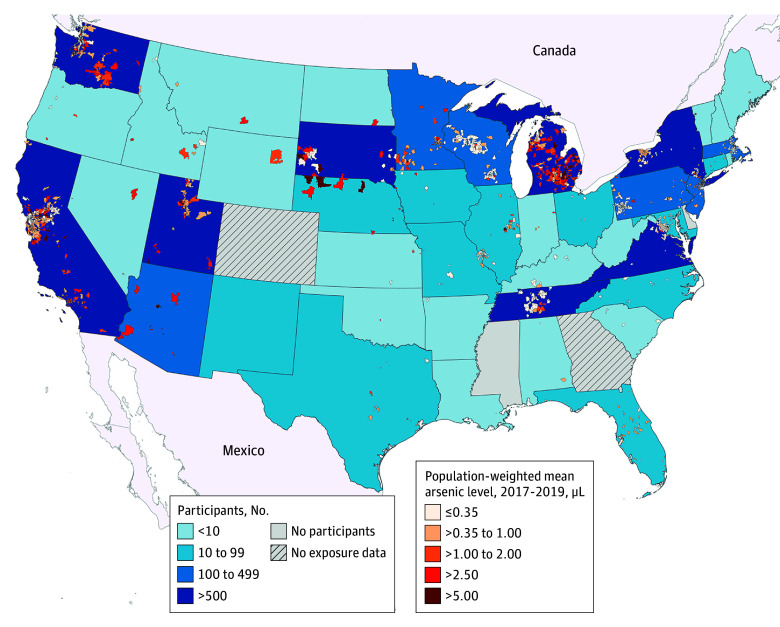
Zip Code Tabulation Area–Level, Population-Weighted, Mean Public Drinking Water Arsenic Concentrations (2017-2019) Across the US Overlaid With the Number of Participants From Each State (N = 13 998) Individual-level, time-weighted prenatal exposure estimates were derived using birthing parent residential address, gestational timing, and Zip Code Tabulated Area–level contaminant concentrations averaged across the 3-year periods (available from 2006 to 2019). Public water arsenic concentration categories correspond to actual and potential regulatory thresholds (5.00 μg/L is the maximum contaminant level for Denmark and the states of New Jersey and New Hampshire; 1.00 μg/L is the maximum contaminant level for the Netherlands) and the modal laboratory limit of detection divided by the square root of 2 (0.35 μg/L). Public water arsenic concentrations were not available in Michigan for the 2017-2019 period; therefore, estimates from 2009 to 2011 (most recently available period) are presented.

The analysis included 13 998 birthing parents (mean [SD] age, 30.8 [5.6] years) of whom 4.5% identified as American Indian, Alaska Native, Native Hawaiian, or Pacific Islander; 7.2% as Asian; 12.4% as Black; 56.1% as White; 4.2% as multiple races; and 8.5% as another race and 28.1% identifying as Hispanic/Latino and 70.4% as non-Hispanic/Latino ethnicity. The participants resided in 1946 ZCTAs within 287 counties and 45 US states, and prenatal public water arsenic concentrations ranged from less than 0.35 to 37.28 μg/L (Figure 1). Prenatal water arsenic was undetectable (<0.35 μg/L) for 7426 participants (53.1%), between 0.35 and 1.00 μg/L for 3529 participants (25.2%), and more than 1.00 μg/L for 3043 participants (21.7%). Of these participants, 236 (1.7%) had water arsenic levels greater than 5.00 μg/L (Table 1). Birthing parents with public water arsenic greater than 5.00 μg/L were more likely to be White (68.6%) and non-Hispanic (87.3%) and to have low-birth-weight infants (7.6%). A total of 1185 births (8.5%) were preterm, 844 (6.0%) were low birth weight, and 1440 (10.3%) were SGA (eTable 1 in Supplement 1).

**Table 1.** Participant Characteristics Overall and Stratified by Prenatal Public Drinking Water Arsenic Concentrations (N = 13 998)

Characteristic	No. (%)					
	Overall	Individual, time-weighted, mean prenatal public water arsenic concentration^a^				
		≤0.35 μg/L	>0.35 to 1.00 μg/L	>1.00 to 2.00 μg/L	>2.00 to 5.00 μg/L	>5.00 μg/L
No. of participants (%)	13 998	7426 (53.1)	3529 (25.2)	1859 (13.3)	948 (6.8)	236 (1.7)
**Birthing parent**						
Age, mean (SD), y	30.8 (5.6)	31.3 (5.6)	30.3 (5.3)	29.7 (5.6)	30.7 (5.4)	30.0 (4.7)
Missing	53 (0.4)	16 (0.2)	5 (0.1)	29 (1.6)	2 (0.2)	1 (0.4)
Race						
American Indian, Alaska Native, Native Hawaiian, or Pacific Islander^b^	623 (4.5)	326 (4.4)	170 (4.8)	28 (1.5)	77 (8.1)	22 (9.3)
Asian	1002 (7.2)	577 (7.8)	302 (8.6)	49 (2.6)	53 (5.6)	21 (8.9)
Black	1729 (12.4)	1081 (14.6)	286 (8.1)	310 (16.7)	40 (4.2)	12 (5.1)
White	7848 (56.1)	3365 (45.3)	2332 (66.1)	1285 (69.1)	704 (74.3)	162 (68.6)
Multiple races	583 (4.2)	285 (3.8)	190 (5.4)	57 (3.1)	42 (4.4)	9 (3.8)
Another race^c^	1193 (8.5)	1052 (14.2)	77 (2.2)	46 (2.5)	<15^d^	<5^d^
Missing	1020 (7.3)	740 (10.0)	172 (4.9)	84 (4.5)	<20^d^	<8^d^
Ethnicity						
Hispanic/Latino	3936 (28.1)	2774 (37.4)	610 (17.3)	433 (23.3)	96 (10.1)	23 (9.7)
Non-Hispanic/Latino	9853 (70.4)	4543 (61.2)	2903 (82.3)	1361 (73.2)	840 (88.6)	206 (87.3)
Missing	209 (1.5)	109 (1.5)	16 (0.5)	65 (3.5)	12 (1.3)	7 (3.0)
Education						
Bachelor’s degree or higher	7173 (51.2)	3671 (49.4)	2104 (59.6)	739 (39.8)	550 (58.0)	109 (46.2)
Some college or associate’s degree	2941 (21.0)	1882 (25.3)	470 (13.3)	451 (24.3)	106 (11.2)	32 (13.6)
Less than high school diploma or equivalent	3262 (23.3)	1564 (21.1)	895 (25.4)	451 (24.3)	266 (28.1)	86 (36.4)
Missing	622 (4.4)	309 (4.2)	60 (1.7)	218 (11.7)	26 (2.7)	9 (3.8)
Prepregnancy BMI, mean (SD)	27.2 (7.0)	26.8 (6.6)	27.5 (7.1)	28.1 (7.5)	27.3 (7.1)	27.3 (7.3)
Missing	1907 (13.6)	1353 (18.2)	225 (6.4)	150 (8.1)	142 (15.0)	37 (15.7)
Prenatal tobacco use						
Yes	680 (4.9)	264 (3.6)	199 (5.6)	139 (7.5)	54 (5.7)	24 (10.2)
No	10 615 (75.8)	5501 (74.1)	2725 (77.2)	1497 (80.5)	702 (74.1)	190 (80.5)
Missing	2703 (19.3)	1661 (22.4)	605 (17.1)	223 (12.0)	192 (20.3)	22 (9.3)
Parity prior to birth						
Multiparous	6879 (49.1)	3186 (42.9)	2065 (58.5)	900 (48.4)	555 (58.5)	173 (73.3)
Nulliparous	4373 (31.2)	2417 (32.5)	1179 (33.4)	421 (22.6)	298 (31.4)	58 (24.6)
Missing	2746 (19.6)	1823 (24.5)	285 (8.1)	538 (28.9)	95 (10.0)	5 (2.1)
Residential location						
Urban	13 349 (95.4)	7219 (97.2)	3358 (95.2)	1776 (95.5)	795 (83.9)	201 (85.2)
Rural	526 (3.8)	166 (2.2)	140 (4.0)	75 (4.0)	115 (12.1)	30 (12.7)
Missing	123 (0.9)	41 (0.6)	31 (0.9)	8 (0.4)	38 (4.0)	5 (2.1)
Socioeconomic vulnerability index score, mean (SD)^e^	0.49 (0.31)	0.53 (0.32)	0.41 (0.27)	0.56 (0.32)	0.38 (0.27)	0.45 (0.27)
Missing	16 (0.1)	9 (0.1)	0	1 (0.1)	5 (0.5)	1 (0.4)
**Infant health outcomes**						
Birth weight, mean (SD), g	3320 (551)	3300 (550)	3340 (545)	3350 (563)	3360 (555)	3290 (570)
Missing	116 (0.8)	52 (0.7)	14 (0.4)	5 (0.3)	37 (3.9)	8 (3.4)
Birth weight–for–gestational age sex-specific z score, mean (SD)	0.04 (1.07)	0.01 (1.07)	0.07 (1.06)	0.09 (1.10)	0.14 (1.03)	−0.03 (1.12)
Missing	117 (0.8)	53 (0.7)	14 (0.4)	5 (0.3)	37 (3.9)	8 (3.4)
Gestational age, mean (SD), wk	38.7 (1.9)	38.6 (1.9)	38.7 (1.9)	38.7 (1.9)	38.7 (2.0)	38.7 (1.8)
Preterm birth^f^	1185 (8.5)	638 (8.6)	293 (8.3)	152 (8.2)	86 (9.1)	16 (6.8)
Low birth weight^g^	844 (6.0)	463 (6.2)	213 (6.0)	105 (5.6)	45 (4.7)	18 (7.6)
Missing	116 (0.8)	52 (0.7)	14 (0.4)	5 (0.3)	37 (3.9)	8 (3.4)
Small for gestational age^g^	1440 (10.3)	812 (10.9)	349 (9.9)	182 (9.8)	71 (7.5)	26 (11.0)
Missing	117 (0.8)	53 (0.7)	14 (0.4)	5 (0.3)	37 (3.9)	8 (3.4)

Abbreviation: BMI, body mass index (calculated as weight in kilograms divided by height in meters squared).

^a^ Prenatal public drinking water arsenic concentrations were assigned via birthing parent residential address during pregnancy. Exposure estimates are individual, time-weighted, pregnancy-period means derived using birthing parent residential address, gestational timing, and Zip Code Tabulation Area–level mean community water system arsenic concentrations available in 3-year periods from 2006 to 2019.

^b^ Combined due to small sample sizes.

^c^ Self-reported race was collected by each cohort site separately; therefore, the racial groups included in the another race category differ by cohort site, and the relevant categories for these participants were unable to be inferred.

^d^ Sample sizes were masked to protect participant confidentiality.

^e^ Centers for Disease Control and Prevention/Agency for Toxic Substances and Disease Registry’s socioeconomic vulnerability score (range, 0-1, with 1 being more vulnerable). Assigned at the census tract level via birthing parent prenatal residential address.

^f^ Defined as less than 37 weeks’ gestation at birth.

^g^ Defined as less than 2500 g at birth.

^g^ Defined as singleton infants with weight less than the 10th percentile of birth weight for gestational age and sex using a 2017 US reference population.

### Prenatal Public Water Arsenic and Birth Outcomes

**Figure 2.**
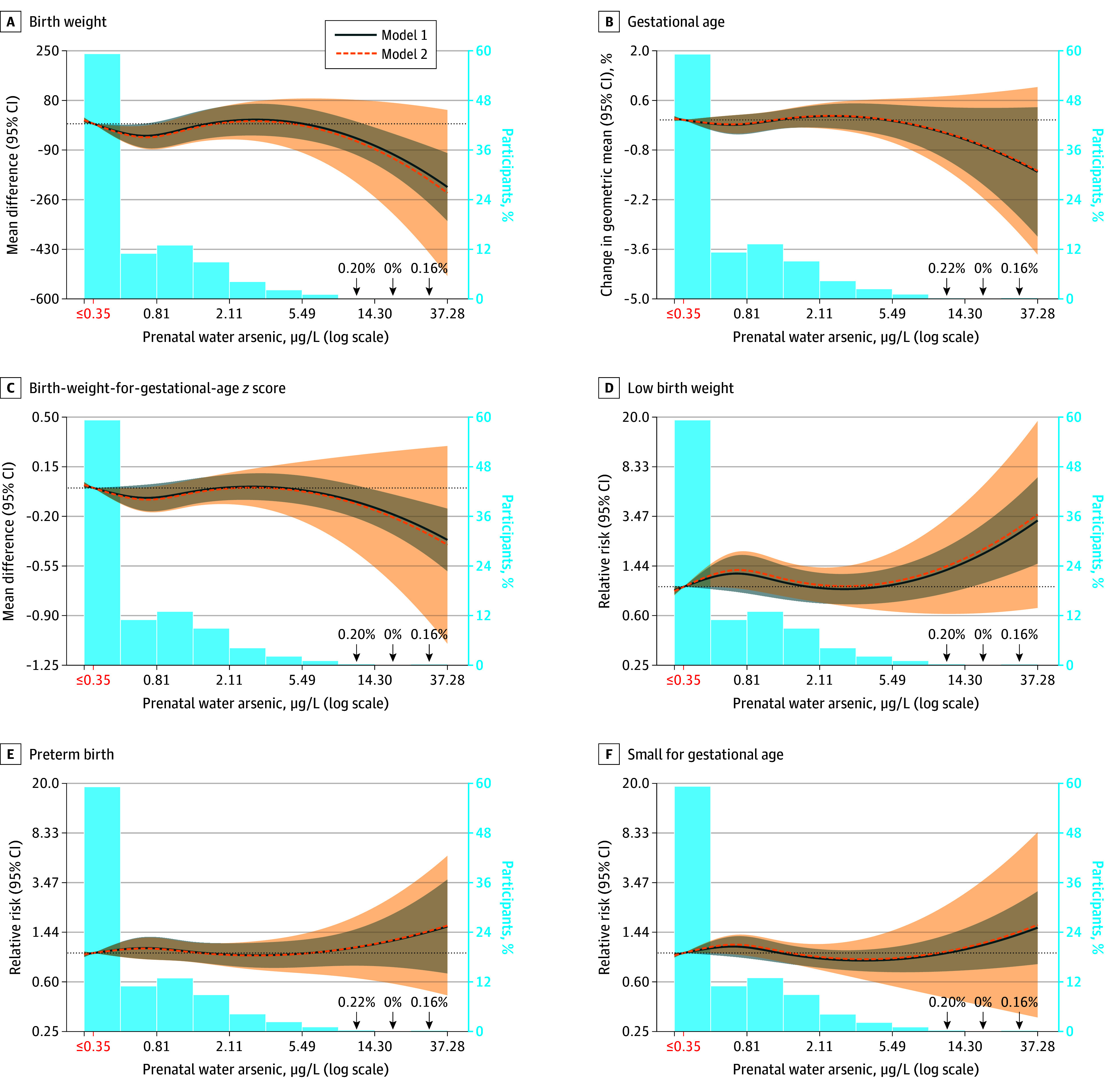
Restricted Cubic Spline Models of the Association Between Prenatal Public Drinking Water Arsenic Exposure and Adverse Birth Outcomes in the Environmental Influences on Child Health Outcomes Cohort (N = 13 998) Models are generalized estimating equations with participants clustered within cohorts (n = 35) using an exchangeable correlation structure. Model 1 was adjusted for birthing parent age (categorical) and education (categorical). Model 2 was additionally adjusted for parity, prepregnancy birthing parent body mass index, prenatal tobacco use, season of conception, and Zip Code Tabulated Area population density. The reference is set to 0.35 μg/L (shown in red, corresponding to undetectable public water arsenic concentrations) with knots at the 67th and 83rd percentiles of the distribution above 0.35 μg/L. Participant prenatal water arsenic concentrations less than 0.35 μg/L represent arsenic concentrations measured below the limit of detection, which were imputed as the limit of detection divided by the square root of 2. Although laboratories reported different limits of detection for different compliance monitoring records, the modal value of the limit of detection divided by the square root of 2 was 0.35 μg/L. The coefficients for the second spline term were −58 (95% CI, −87 to −28) for birth weight; 0% for gestational age; −0.10 (95% CI, −0.16 to −0.03) for birth weight–for–gestational age *z* score; 1.39 (95% CI, 1.10-1.76) for low birth weight; 1.12 (95% CI, 0.92-1.36) for preterm birth; and 1.22 (95% CI, 1.07-1.40) for small for gestational age.

In adjusted flexible cubic spline models, higher prenatal public water arsenic was associated with lower birth weight, reduced birth weight–for–gestational age *z* score, and a higher risk of low birth weight even at concentrations less than 5.00 μg/L (Figure 2). Across all outcomes, precision was limited by the relatively small number of participants with prenatal arsenic greater than 5.00 μg/L. Findings were consistent with alternative knots (eFigure 2 in Supplement 1).

**Figure 3.**
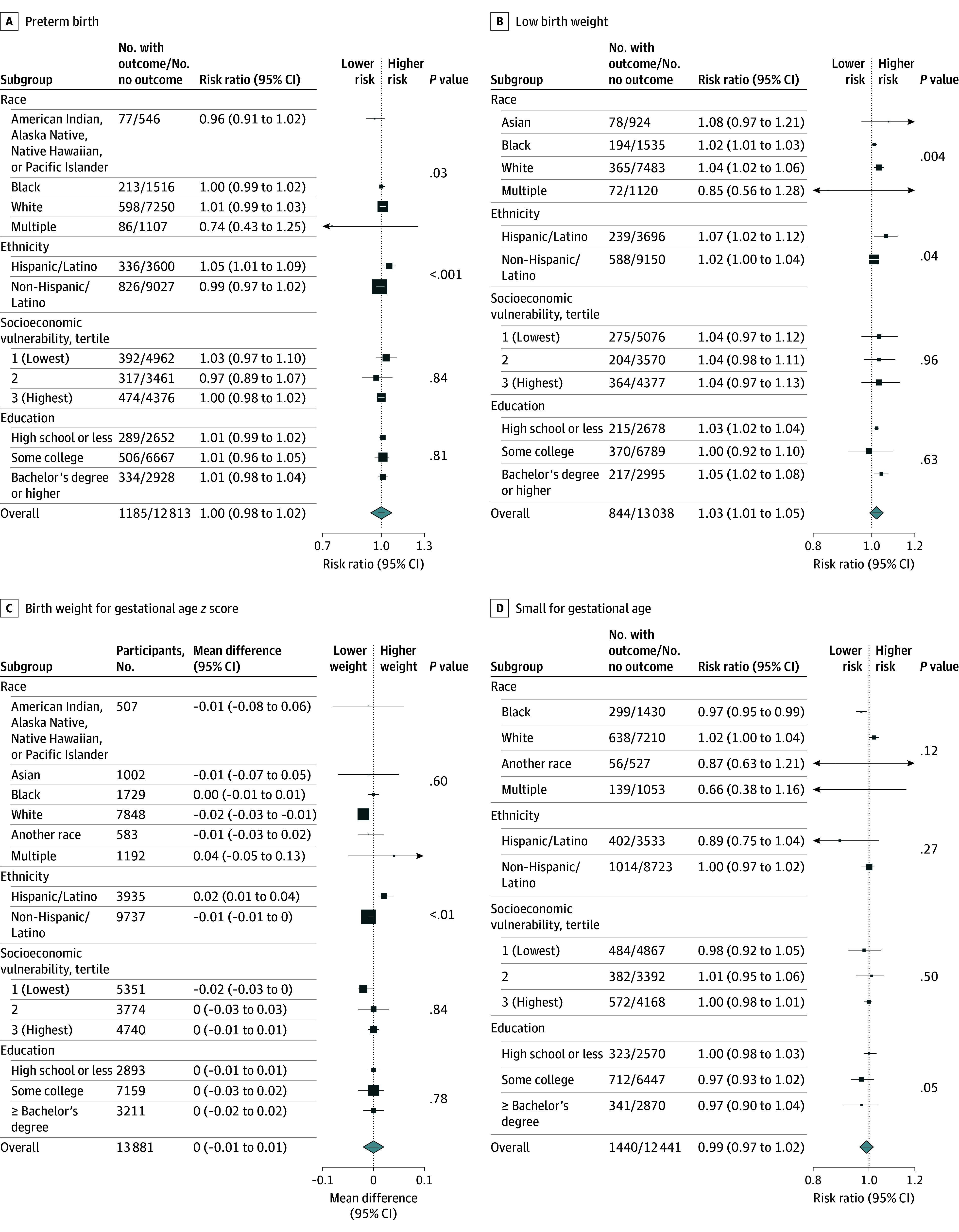
Associations Between Prenatal Public Water Arsenic Exposure and Birth Outcomes in the Environmental Influences on Child Health Outcomes Cohort in Stratified Subgroup Analyses (N = 13 998) Associations represent effect estimates per a 1.00 μg/L higher prenatal public water arsenic exposure in linear models (model 2 adjustments). Square size indicates the number of participants in each subgroup. Effect estimates were not reported for subgroups with less than 50 events or when models did not converge because of small sample/case size. Wald test *P* values reflect statistical interactions across all strata, including those with fewer than 50 cases and when models did not converge.

Overall, the RRs of low birth weight per 1.00 and 5.00 μg/L higher prenatal public water arsenic was 1.03 (95% CI, 1.01-1.05) and 1.16 (95% CI, 1.05-1.28), respectively (Figure 3; eTable 2 in Supplement 1). Effect estimates differed by birthing parent subgroup. The RR for low birth weight per 1.00 and 5.00 μg/L was higher among Black (1.02 [95% CI, 1.01-1.03] and 1.10 [95% CI, 1.05-1.16], respectively), Hispanic/Latino (1.07 [95% CI, 1.02-1.12] and 1.39 [95% CI, 1.09-1.75], respectively), and White (1.04 [95% CI, 1.02-1.06] and 1.22 [95% CI, 1.10-1.34], respectively) birthing parents (*P* for interaction = .004) (Figure 3). Prenatal public water arsenic (per 1.00 μg/L) was associated with birth weight–for–gestational age *z* score mean difference of −0.02 (95% CI, −0.03 to −0.01), SGA RR of 1.02 (95% CI, 1.00-1.04), and birth weight mean difference of –10 g (95% CI, –7 to –3 g) among White birthing parents and with preterm birth for Hispanic/Latino birthing parents (RR, 1.05 [95% CI, 1.01-1.09]). We did not observe a consistent pattern of association across birthing parent education or socioeconomic vulnerability.

In categorical analyses comparing prenatal public water arsenic greater than 5.00 μg/L with less than 0.35 μg/L, the effect estimates were larger in magnitude for all outcomes compared with continuous models, although sample sizes were very small and estimates lacked precision (eTable 2 in Supplement 1). Specifically, the RRs of low birth weight and SGA were 1.46 (95% CI, 0.85-2.48) and 1.22 (95% CI, 0.90-1.65), respectively, and the mean difference in birth weight and birth weight–for–gestational age *z* score were –51 g (95% CI, –118 to 15 g) and −0.16 SDs (95% CI, −0.29 to −0.03 SDs), respectively. We did not observe a clear exposure-response association in categorical models. Interpretations were similar when considering all alternative model adjustments, g-computation, and exclusion criteria (eTable 3 in Supplement 1). Excluding 1 cohort in Michigan (MARCH) in which water arsenic levels were relatively high resulted in attenuated results for most outcomes (eFigure 3 in Supplement 1). Findings were largely similar when using census tract–level exposures, with some attenuated effect estimates for White participants (eFigures 4 and 5 and eTable 4 in Supplement 1). A directed acyclic graphic is available in eFigure 6 in Supplement 1.

### Racial and Ethnic Disparities in Adverse Birth Outcomes

Table 2 presents disparities in birth outcomes by birthing parent race, ethnicity, and race and ethnicity and estimated mediation effects. Compared with non-Hispanic/Latino birthing parents, infants born to Hispanic/Latino birthing parents had higher birth weights and were less likely to be born low birth weight and SGA. Compared with White birthing parents, infants born to Black birthing parents had lower mean birth weight (mean difference, −245 g [95% CI, −283 to −206 g]) and a higher risk of preterm birth (RR, 1.48 [95% CI, 1.22-1.80]), low birth weight (RR, 2.35 [95% CI, 1.88-2.94]), and SGA (RR, 2.15 [95% CI, 1.75-2.64]). Infants born to American Indian, Alaska Native, Native Hawaiian, or Pacific Islander birthing parents also had a higher risk of preterm birth (RR, 1.37 [95% CI, 1.09-1.72]) and low birth weight (RR, 1.33 [95% CI, 1.01-1.76]). Compared with non-Hispanic White birthing parents, infants born to parents in the all other classification had lower birth weights (mean difference, −118 g [95% CI, −154 to −82 g]) and a higher risk of preterm birth (RR, 1.26 [95% CI, 1.08-1.47]), low birth weight (RR, 1.61 [95% CI, 1.28-2.03]), and SGA (RR, 1.54 [95% CI, 1.37-1.73]). We observed no evidence of mediation by prenatal public water arsenic.

**Table 2.** Racial, Ethnic, and Racial and Ethnic Disparities in Birth Outcomes in the ECHO Cohort (N = 13 998)

Variable	Mean difference (95% CI)		Gestational age change in GMR, % (95% CI)	RR (95% CI)		
	Birth weight, g	BWGA *z* score		Preterm birth (<37 wk)	Low birth weight (<2500 g)	SGA^a^
**Disparities by birthing parent ethnicity**						
Hispanic/Latino vs non-Hispanic/Latino (reference)						
Model 1	41 (1 to 80)	0.06 (−0.02 to 0.14)	0.3 (0.0 to 0.5)	0.91 (0.78 to 1.07)	0.82 (0.64 to 1.05)	0.88 (0.75 to 1.03)
Natural indirect effect^b^	−0 (−28 to 28)	0.00 (−0.06 to 0.05)	0.0 (−0.3 to 0.3)	1.00 (0.84 to 1.18)	0.99 (0.81 to 1.22)	1.01 (0.87 to 1.18)
**Disparities by birthing parent race**						
All races other than White vs White (reference)						
Model 1	−126 (−164 to −88)	−0.24 (−0.33 to −0.16)	−0.4 (−0.7 to −0.2)	1.19 (1.04 to 1.37)	1.66 (1.40 to 1.95)	1.62 (1.43 to 1.84)
Natural indirect effect^b^	1 (−25 to 28)	0.00 (−0.05 to 0.05)	0.0 (−0.2 to 0.3)	1.00 (0.85 to 1.16)	0.99 (0.82 to 1.19)	1.00 (0.87 to 1.15)
Black vs White (reference)						
Model 1	−245 (−283 to −206)	−0.46 (−0.55 to −0.37)	−1.0 (−1.6 to −0.4)	1.48 (1.22 to 1.80)	2.35 (1.88 to 2.94)	2.15 (1.75 to 2.64)
Natural indirect effect^b^	1 (−39 to 42)	0.00 (−0.08 to 0.08)	0.0 (−0.4 to 0.4)	1.00 (0.82 to 1.22)	0.99 (0.79 to 1.25)	1.00 (0.84 to 1.19)
American Indian, Alaska Native, Native Hawaiian, or Pacific Islander vs White (reference)						
Model 1	42 (−30 to 114)	0.14 (−0.04 to 0.31)	−0.2 (−0.6 to 0.2)	1.37 (1.09 to 1.72)	1.33 (1.01 to 1.76)	1.07 (0.78 to 1.47)
Natural indirect effect^b^	1 (−68 to 69)	0.00 (−0.14 to 0.14)	0.0 (−0.6 to 0.6)	1.00 (0.74 to 1.36)	1.00 (0.62 to 1.59)	1.00 (0.66 to 1.51)
**Disparities by birthing parent race and ethnicity**						
Hispanic/Latino and/or not White vs both non-Hispanic/Latino and White (reference)						
Model 1	−118 (−154 to −82)	−0.22 (−0.30 to −0.15)	−0.5 (−0.7 to −0.2)	1.26 (1.08 to 1.47)	1.61 (1.28 to 2.03)	1.54 (1.37 to 1.73)
Natural indirect effect^b^	1 (−25 to 27)	0.00 (−0.05 to 0.05)	0.0 (−0.2 to 0.3)	1.00 (0.85 to 1.17)	0.99 (0.81 to 1.20)	1.00 (0.87 to 1.16)

Abbreviations: BWGA, birth weight for gestational age; ECHO, Environmental Influences on Child Health Outcomes; GMR, geometric mean ratio; RR, risk ratio; SGA, small for gestational age.

^a^ Defined as singleton infants with weight less than the 10th percentile of BWGA and sex using a 2017 US reference population.^17^

^b^ The effect on the outcome when the exposure is held fixed but the mediator value is changed as it would have been changed if the exposure value were increased by 1 unit. Prenatal public water arsenic exposure was assigned using birthing parent residential address history during pregnancy as individual, time-weighted concentrations from Zip Code Tabulation Area–level, population-weighted mean concentrations. Models are generalized estimating equations with participants clustered within cohorts (n = 35) using an exchangeable correlation structure. Missingness for all covariates was imputed via multiple imputation with chained equations (5 iterations and 10 imputed datasets). Model 1 was adjusted for birthing parent age (categorical) and education (categorical), infant sex, parity, season of conception, prepregnancy body mass index (centered), prenatal tobacco use, and Zip Code Tabulation Area population density (centered) (directed acyclic graph available in eFigure 6 in Supplement 1). Indirect effects were estimated from the GEEmediate package in R, version 4.4.0; in all models, no total or direct effects were estimated, indicating no mediation by public drinking water arsenic.

## Discussion

In this cohort study of 35 pooled pregnancy cohorts across the US, higher prenatal public water arsenic was associated with decreased birth weight and birth weight–for–gestational age *z* score and an increased risk of low birth weight at water arsenic concentrations below the EPA MCL of 10 μg/L. Associations were also suggestive for preterm birth among Hispanic/Latino birthing parents and SGA. Findings were generally similar to prior studies of private wells, higher exposures, and single states.^3,6,7,10,27^ The EPA recently concluded that “evidence indicates” that inorganic arsenic is likely causally associated with length of gestation and birth weight, although the current epidemiologic evidence at low arsenic concentrations in regulated public water systems is limited.^5^ Our findings in the ECHO Cohort further support that the current EPA MCL is inadequately protective of adverse birth outcomes.^2^ These findings could be used to support individual, household, and community-level exposure reduction interventions, including participatory household filtration programs^28^ and clinic-based prenatal household water testing programs. In New Jersey communities with relatively high groundwater arsenic concentrations, prior interventions have leveraged health care appointments and electronic medical records to offer household water arsenic testing to patients using private wells.^29^ US public drinking water arsenic is highest among rural communities, in the Southwest and Midwest, and for American Indian or Alaska Native and Hispanic/Latino communities.^14,26^ In these communities, primary and prenatal care appointments are potential opportunities for arsenic education and household water testing initiatives, even for patients connected to regulated public systems.^29^

We identified adverse birth outcome disparities by race and ethnicity within the ECHO Cohort. Despite consistent evidence that public drinking water arsenic is highest for American Indian or Alaska Native and Hispanic/Latino residents across the US, we did not observe these disparities in ECHO and found no evidence that public drinking water arsenic mediated outcome disparities in the ECHO Cohort. The majority of ECHO participants reside in urban areas, and while the ECHO Cohort is geographically, racially, and ethnically diverse, it may not be representative of the general US population (8.5% of births in our sample were preterm compared with 10.4% nationwide in 2021), and our sample size was limited for binary outcomes.^12^ Additional studies leveraging vital and/or electronic health records are an opportunity to overcome these limitations. We identified some differences in the association stratified by race and ethnicity, which may reflect either differential environmental or social exposures across groups that influence the association (differential effects of structural racism), differential measurement error of the exposure (especially relevant for water hauling) (eMethods in Supplement 1), or insufficient sampling of participants from some racial and ethnic groups from all neighborhood types.^30^ We were unable to evaluate nonlinear models in subgroup analyses because of small sample sizes and case numbers; therefore, effect estimates from these linear models may not appropriately represent associations occurring within each subgroup.

### Limitations

Our study had several limitations. The sample included a small number of participants with water arsenic exposure greater than 5.00 μg/L. Although more than 94% of US residents receive at least some water from regulated community water systems that serve the same populations year round,^31^ self-reported tap water source was only available for a subset of participants (8.5%), and we did not have adequate data to assess whether participants consumed tap vs bottled water. We assigned public water arsenic concentrations at the ZCTA- and census tract–level, but it is not yet clear whether water system-, census tract–, ZCTA-, or other area-level estimates best reflect total public water exposures from primary (residential) and secondary (eg, work, school) sources. The ZCTA-level arsenic exposure estimates assume that participants only consume water from systems within their residential ZCTAs and do not account for secondary exposures occurring at places of work, school, worship, or play outside of residential ZCTAs.^13^ Our current effect estimates were likely influenced by exposure measurement error, especially in states with poorly characterized water system distribution boundaries. Further research is needed to robustly characterize exposure measurement error in public drinking water exposure assessment and epidemiologic studies. Measurement error of the exposure is a particular concern at the lower ends of the exposure distribution, which may explain the shape of the exposure-response function observed in our spline models. Because arsenic is associated with miscarriage and stillbirth at high concentrations, live birth bias may also be relevant.^4^ Future analyses should consider non–birthing parent/caregiver characteristics in analyses of outcome disparities and potential mediation by water contaminants. We did not evaluate other sources of inorganic arsenic (rice consumption) that may be more relevant for some cohort sites and differential by race and ethnicity. Relevant dietary and supplement data (eg, folate, vitamin B_12_) was not harmonized or available for analysis.

## Conclusions

In this cohort study of birthing parent–infants dyads from 35 ECHO Cohort sites, arsenic measured in public water systems was associated with birth outcomes at levels below the current EPA’s MCL. Public drinking water contaminants are directly relevant for child health and are readily modifiable by federal regulatory action, yet they remain underappreciated and understudied in the environmental epidemiology literature.^2^ Population-, community-, household-, and individual-level exposure reduction interventions can be leveraged to reduce water arsenic exposure for all US communities. Additional federal financial, technical, and managerial support is urgently needed to reduce arsenic concentrations in regulated public drinking water systems, many of which continue to exceed the EPA MCL of 10 μg/L.^14^

## Notes

Supplement 1. **eFigure 1.** Exclusion Criteria **eMethods.** Detailed Description of Exposure Assessment and Statistical Analysis **eTable 1.** Participant Characteristics Overall and Stratified by Categorical Adverse Infant Birth Outcomes **eTable 2.** Associations Between Prenatal Public Water Arsenic Exposure and Birth Outcomes in the ECHO Cohort (N = 13 998) With Arsenic Exposure Available **eTable 3.** Sensitivity Analyses Evaluating the Association Between a 1 μg/L Higher Prenatal Public Water Arsenic Exposure and Birth Outcomes in the ECHO Cohort, Considering Alternative Model Adjustments and Restriction Criteria **eFigure 2.** Restricted Cubic Spline Models of the Association Between Prenatal Public Drinking Water Arsenic Exposure and Adverse Birth Outcomes in the ECHO Cohort (N = 13 998), With Alternative Knot Locations at the 60th and 90th Percentiles **eFigure 3.** Results of Sensitivity Analysis Removing Each Individual Cohort (Leave One Out) to Identify Influential Chorts in the ECHO Cohort **eFigure 4.** Restricted Cubic Spline Models of the Association Between Prenatal Public Drinking Water Arsenic Exposure and Adverse Birth Outcomes in the ECHO Cohort When Evaluating Public Water Arsenic at the Tract Level **eTable 4.** Sensitivity Analyses Evaluating the Association Between Higher Prenatal Public Water Arsenic Exposure and Birth Outcomes in the ECHO Cohort, Using Tract-Level Exposure Estimates **eFigure 5.** Sensitivity Analyses Evaluating the Association Between Higher Prenatal Public Water Arsenic Exposure and Birth Outcomes in the ECHO Cohort, Using Tract-Level Exposure Estimates **eFigure 6.** Directed Acyclic Graph
eReferences.

Supplement 2.
Nonauthor Contributors

Supplement 3.
Data Sharing Statement
