# Supplementary material for: Public Water Arsenic and Birth Outcomes in the Environmental Influences on Child Health Outcomes Cohort
Source: JAMA Netw Open. 2025 Jun 16;8(6):e2514084. doi: 10.1001/jamanetworkopen.2025.14084 (PMC12171937; doi:10.1001/jamanetworkopen.2025.14084)
Supplement: Supplement 1. — eFigure 1. Exclusion Criteria eMethods. Detailed Description of Exposure Assessment and Statistical Analysis eTable 1. Participant Characteristics Overall and Stratified by Categorical Adverse Infant Birth Outcomes eTable 2. Associations Between Prenatal Public Water Arsenic Exposure and Birth Outcomes in the ECHO Cohort (N = 13 998) With Arsenic Exposure Available eTable 3. Sensitivity Analyses Evaluating the Association Between a 1 μg/L Higher Prenatal Public Water Arsenic Exposure and Birth Outcomes in the ECHO Cohort, Considering Alternative Model Adjustments and Restriction Criteria eFigure 2. Restricted Cubic Spline Models of the Association Between Prenatal Public Drinking Water Arsenic Exposure and Adverse Birth Outcomes in the ECHO Cohort (N = 13 998), With Alternative Knot Locations at the 60th and 90th Percentiles eFigure 3. Results of Sensitivity Analysis Removing Each Individual Cohort (Leave One Out) to Identify Influential Chorts in the ECHO Cohort eFigure 4. Restricted Cubic Spline Models of the Association Between Prenatal Public Drinking Water Arsenic Exposure and Adverse Birth Outcomes in the ECHO Cohort When Evaluating Public Water Arsenic at the Tract Level eTable 4. Sensitivity Analyses Evaluating the Association Between Higher Prenatal Public Water Arsenic Exposure and Birth Outcomes in the ECHO Cohort, Using Tract-Level Exposure Estimates eFigure 5. Sensitivity Analyses Evaluating the Association Between Higher Prenatal Public Water Arsenic Exposure and Birth Outcomes in the ECHO Cohort, Using Tract-Level Exposure Estimates eFigure 6. Directed Acyclic Graph eReferences. [file jamanetwopen-e2514084-s001.pdf]

Supplemental Online Content

Nigra AE, Bloomquist TR, Rajeev T, et al. Public water arsenic and birth outcomes in the Environmental Influences on Child Health Outcomes Cohort. *JAMA Netw Open*. 2025;8(6):e2514084. doi:10.1001/jamanetworkopen.2025.14084

eFigure 1. Exclusion Criteria

eMethods. Detailed Description of Exposure Assessment and Statistical Analysis

eTable 1. Participant Characteristics Overall and Stratified by Categorical Adverse Infant Birth Outcomes

eTable 2. Associations Between Prenatal Public Water Arsenic Exposure and Birth Outcomes in the ECHO Cohort (N = 13 998) With Arsenic Exposure Available

eTable 3. Sensitivity Analyses Evaluating the Association Between a 1 µg/L Higher Prenatal Public Water Arsenic Exposure and Birth Outcomes in the ECHO Cohort, Considering Alternative Model Adjustments and Restriction Criteria

eFigure 2. Restricted Cubic Spline Models of the Association Between Prenatal Public Drinking Water Arsenic Exposure and Adverse Birth Outcomes in the ECHO Cohort (N = 13 998), With Alternative Knot Locations at the 60th and 90th Percentiles

eFigure 3. Results of Sensitivity Analysis Removing Each Individual Cohort (Leave One Out) to Identify Influential Chorts in the ECHO Cohort

eFigure 4. Restricted Cubic Spline Models of the Association Between Prenatal Public Drinking Water Arsenic Exposure and Adverse Birth Outcomes in the ECHO Cohort When Evaluating Public Water Arsenic at the Tract Level

eTable 4. Sensitivity Analyses Evaluating the Association Between Higher Prenatal Public Water Arsenic Exposure and Birth Outcomes in the ECHO Cohort, Using Tract-Level Exposure Estimates

eFigure 5. Sensitivity Analyses Evaluating the Association Between Higher Prenatal Public Water Arsenic Exposure and Birth Outcomes in the ECHO Cohort, Using Tract-Level Exposure Estimates

eFigure 6. Directed Acyclic Graph

eReferences

This supplemental material has been provided by the authors to give readers additional information about their work.

© 2025 Nigra AE et al. *JAMA Network Open*.

eFigure 1. Exclusion Criteria. To align exposure data with prenatal periods, we excluded births missing exposure data for >50% of the pregnancy period (this resulted in the exclusion of births occurring prior to 2005).

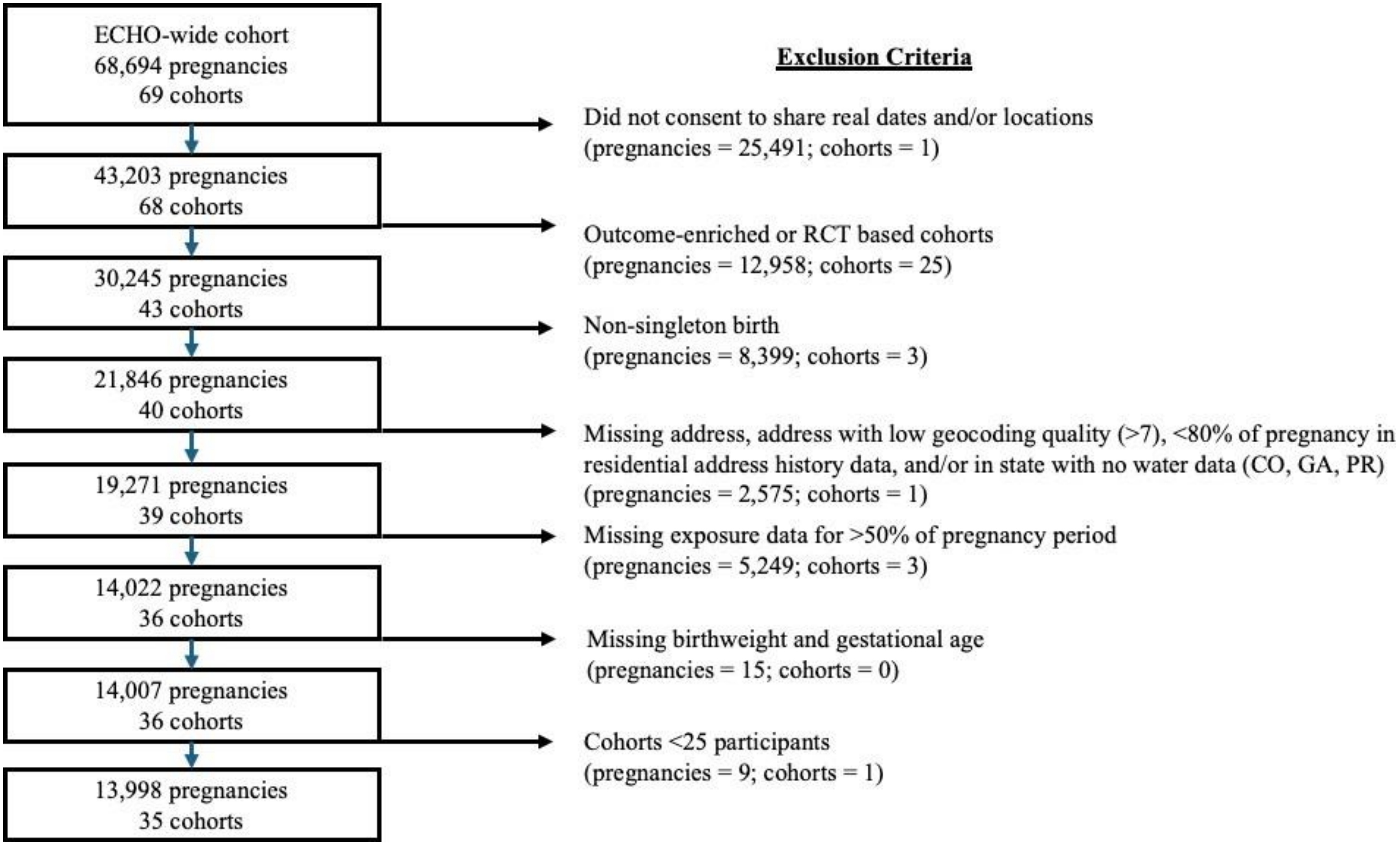

© 2025 Nigra AE et al. *JAMA Network Open*.

eMethods. Detailed Description of Exposure Assessment and Statistical Analysis

Zip Code Tabulation Area (ZCTA)- and tract- level public water arsenic concentrations.

Detailed methods describing the development of area-level (ZCTA and tract) public water arsenic concentrations have been previously published.[1] Briefly, we leveraged previously developed ZCTA-level estimates of average, population-weighted public water arsenic concentrations, which were previously validated in two multi-site cohorts with urinary biomarkers reflecting total internal dose.[1,2] Briefly, these ZCTA-level estimates were generated by aggregating community water system arsenic concentrations to 2010 ZCTA-boundaries and applying population weights. The previously generated community water system arsenic concentrations were developed from >600,000 routine compliance monitoring records collected by >37,000 community water systems, which were collected by the USEPA for the Contaminant Occurrence Database supporting the Third (covering years 2006-2011) and Fourth (covering years 2012-2019) Six Year Review. The USEPA's Third and Fourth Six Year Review databases are the most recently available nationwide dataset of routine compliance monitoring records, representing >95% of all public water systems nationwide, and are the most extensive database on public drinking water contaminant occurrence that USEPA has ever collected.[3-5] USEPA conducted extensive quality control and data cleaning prior to publishing the Six Year Review databases.[4-6] To reduce differential missingness and bias,[3] community water system and ZCTA-level estimates were aggregated to three-year time periods corresponding with the USEPA's Standardized Monitoring Framework[7] time periods (e.g., 2014-2016, 2017-2019).[3,8,9] Water system level concentrations reflect concentrations distributed to consumers in finished water (i.e., after any potential treatment at the utility).[3]

Community water systems can serve multiple ZCTAs/tracts and ZCTAs/tracts can be served by multiple community water systems. We leveraged nationwide community water system service boundaries modeled and published by USEPA.[10,11] Water systems were assigned to ZCTAs/tracts by intersecting ZCTA/tract boundaries with water system service boundaries.[1] Population-weighted, ZCTA-level public water arsenic concentrations were previously validated with urinary biomarkers (reflecting total internal dose) measured in 8,000 participants from two NIH-funded, multi-site cohorts which included both urban and rural areas.[2]

Although compliance monitoring records reported different analytical limits of detection, USEPA sets a minimum reporting level at 0.5 µg/L. Before aggregating to the ZCTA/tract-level, we imputed water system arsenic values less than the record specific detection limit as the detection limit

divided by the square root of two. As such, ZCTA/tract-level estimates less than or equal to 0.35 µg/L reflect either (a) arsenic concentrations measured below the limit of detection, or (b) very low arsenic concentrations measured above the detection limit at laboratories with unusually high precision and low detection limits.

Individual participant-level prenatal public water uranium concentrations were estimated similarly, although these were aggregated to the 2000-2011 period to account for differences in the Standard Monitoring Framework under the Radionuclides Rule.[4,12] We adjusted for uranium concentrations in sensitivity analyses because arsenic is most highly correlated with uranium in public water systems (rho=0.25).[12] We could not assign ZCTA/tract-level public water arsenic estimates to birthing parents from one cohort because participants often relied on hauling water.[13] We assigned these participants to public water system level estimates[3] according to self-reported drinking water source (which was previously matched to public water systems),[13] and excluded these participants in sensitivity analyses.

ZCTA/tract-level estimates were aggregated to three-year time periods corresponding with the USEPA's Standardized Monitoring Framework[7] time periods (e.g., 2014-2016, 2017-2019), reflecting USEPA compliance monitoring periods accompanying the Final Arsenic Rule.[14] ZCTA/tract-level uranium concentrations were developed following this same approach, and were aggregated to the 2000-2011 time period to reflect the Radionuclides Rule compliance monitoring period.[12] To develop individual-level, time-weighted, average prenatal public water arsenic, each pregnancy month was assigned the overlapping ZCTA-level arsenic average. The state of Michigan reported arsenic monitoring records during the Third Six Year Review (2006-2011) but did not report records for the Fourth Six Year Review (2012-2019).[15] Therefore, for pregnancies occurring in Michigan after 2011, we carried forward estimates from the 2009-2011 time period for all pregnancy months occurring after 2011. Participants in one cohort reported reliance on water hauling from regulated water sources. For a prior project, we matched participant identified water sources to regulated public drinking water systems.[13] We were therefore able to match participants from this cohort directly to individual public water systems rather than relying on ZCTA-level averages. To evaluate whether this differential exposure assessment impacted our overall findings, we removed participants from this cohort from analysis, with similar findings (see eFigure 4). However, we anticipate there may

be some differential measurement error in the exposure by rural vs urban status and potentially by birthing parent race and ethnicity because of differential measurement error in estimated USEPA community water

© 2025 Nigra AE et al. *JAMA Network Open*.

system boundaries estimates. Such differential error results from some states publishing high-quality service area shapefiles, while other service area boundaries are solely based on modeled boundaries by USEPA.[11]

Covariates

Individual-level covariates included infant sex (female / male [ref]), conception season (categorical: spring [March, April, May] / summer [June, July, August] [ref] / fall [September, October, November] / winter [December, January, February]), birthing parent education (≤ high school degree or equivalent / some college or Associate's degree / Bachelor's degree or higher [ref]), age at delivery (<25 [ref] / 25-29 / 30-34 / ≥35), pre-pregnancy body mass index (BMI) (continuous, centered), prenatal tobacco use (any / none [ref]), parity (nulliparous [ref] / multiparous), birthing parent race (combined as American Indian, Alaskan Native, Native Hawaiian, or Pacific Islander due to small sample sizes / Another race / Asian / Black / Multiple races / White) and ethnicity (Hispanic / non-Hispanic), and prenatal public water uranium estimates (continuous, µg/L). Area-level covariates included ZCTA-level population density (population divided by land area in square miles, centered) and census tract socioeconomic vulnerability index score (continuous, higher scores indicate higher socioeconomic vulnerability). The socioeconomic vulnerability index score was developed by the Centers for Disease Control and Prevention/Agency for Toxic Substances and Disease Registry (CDC/ATSDR) derived from US Census American Community Survey estimates of median household income and the percent of adults who are unemployed, living below the poverty line, and without a high school diploma.[16] Data from the 2010, 2014, 2016, 2018, and 2020 datasets were utilized in this analysis.

Statistical analysis

We used Poisson models to evaluate the risk ratio (RR) of binary outcomes because this approach allowed us to calculate risk ratios rather than odds ratios, Poisson models with robust variance can robustly model binary outcome data even in longitudinal settings,[17] and because of convergence issues that are common within ECHO when logistic regression approaches attempt to incorporate either generalized estimating equation (GEE) models or mixed effects models with multicenter data.[18, 19] For all models, we used multiple imputation by chained equations (MICE, n=5 iterations and 25 imputed datasets) to impute

© 2025 Nigra AE et al. *JAMA Network Open*.

missing covariates. Missingness ranged from 0.4% (birthing parent age) to 19.6% (parity) for individual-level covariates and from 0.1% (socioeconomic vulnerability index) to 50.9% (uranium) for area-level covariates.

© 2025 Nigra AE et al. *JAMA Network Open*.

eTable 1. Participant Characteristics Overall and Stratified by Categorical Adverse Infant Birth Outcomes (preterm birth, low birthweight, and small and large for gestational age, n= 13,998).

	Overall (N=13,998)	Preterm birth, <37 weeks (n=1,185, 8.5%)	Low birthweight (<2,500 g) (n=844, 6.0%)	Small for gestational age [b] (n=1,440, 10.3%)
Birthing parent characteristics				
Age (mean, SD)	30.8 (5.55)	31.2 (6.04)	31.1 (6.08)	30.3 (5.88)
Missing (n, %)	53 (0.4%)	4 (0.3%)	1 (0.1%)	2 (0.1%)
Race (n, %)				
AI/ AN/ NH/ PI [a]	623 (4.5%)	77 (6.5%)	32 (3.8%)	37 (2.6%)
Another Race	1193 (8.5%)	86 (7.3%)	72 (8.5%)	139 (9.7%)
Asian	1002 (7.2%)	82 (6.9%)	78 (9.2%)	154 (10.7%)
Black	1729 (12.4%)	213 (18.0%)	194 (23.0%)	299 (20.8%)
Multiple Races	583 (4.2%)	43 (3.6%)	36 (4.3%)	56 (3.9%)
White	7848 (56.1%)	598 (50.5%)	365 (43.2%)	638 (44.3%)
Missing	1020 (7.3%)	86 (7.3%)	67 (7.9%)	117 (8.1%)
Ethnicity (n, %)				
Hispanic	3936 (28.1%)	336 (28.4%)	239 (28.3%)	402 (27.9%)
non-Hispanic	9853 (70.4%)	826 (69.7%)	588 (69.7%)	1014 (70.4%)
Missing	209 (1.5%)	23 (1.9%)	17 (2.0%)	24 (1.7%)
Education (n, %)				
≥Bachelor's deg.	7173 (51.2%)	506 (42.7%)	370 (43.8%)	712 (49.4%)
Some college or Associate's deg.	2941 (21.0%)	289 (24.4%)	215 (25.5%)	323 (22.4%)
≤ High school deg. or equivalent	3262 (23.3%)	334 (28.2%)	217 (25.7%)	341 (23.7%)
Missing	622 (4.4%)	56 (4.7%)	42 (5.0%)	64 (4.4%)
Pre-pregnancy BMI (mean, SD)	27.2 (6.95)	28.2 (7.66)	26.8 (6.60)	25.9 (6.24)
Missing	1907 (13.6%)	169 (14.3%)	112 (13.3%)	208 (14.4%)
Prenatal tobacco use (n, %)				
Yes	680 (4.9%)	74 (6.2%)	49 (5.8%)	73 (5.1%)
No	10615 (75.8%)	868 (73.2%)	617 (73.1%)	1028 (71.4%)
Missing	2703 (19.3%)	243 (20.5%)	178 (21.1%)	339 (23.5%)
Parity prior to birth ≥1 (n, %)				
Multiparous	6879 (49.1%)	599 (50.5%)	370 (43.8%)	527 (36.6%)
Nulliparous	4373 (31.2%)	349 (29.5%)	284 (33.6%)	568 (39.4%)
Missing	2746 (19.6%)	237 (20.0%)	190 (22.5%)	345 (24.0%)
Urban location (n, %)				
Urban	13349 (95.4%)	1123 (94.8%)	820 (97.2%)	1400 (97.2%)
Rural	526 (3.8%)	46 (3.9%)	17 (2.0%)	31 (2.2%)
Missing	123 (0.9%)	16 (1.4%)	7 (0.8%)	9 (0.6%)
Socioeconomic vulnerability index score [c] (mean, SD)	0.492 (0.311)	0.531 (0.313)	0.543 (0.314)	0.528 (0.312)
Missing (n, %)	16 (0.1%)	2 (0.2%)	1 (0.1%)	2 (0.1%)
Prenatal public water metal exposures, µg/L (mean, SD)				
Arsenic	0.920 (1.68)	0.946 (1.93)	0.985 (2.42)	0.855 (1.62)
Barium	36.6 (69.4)	38.6 (88.8)	32.4 (58.0)	34.3 (51.1)
Chromium	0.861 (1.79)	0.733 (1.51)	0.678 (1.50)	0.837 (1.54)
Selenium	0.932 (1.04)	0.925 (1.03)	1.01 (1.10)	0.989 (1.04)
Uranium	3.68 (5.18)	3.42 (4.93)	3.37 (4.83)	3.12 (4.71)

BMI = body mass index.

[a] American Indian/ Alaskan Native/ Native Hawaiian/ Pacific Islander (combined due to small sample sizes)

[b] Defined a singleton infants with weight <10th percentile of birthweight-for-gestational-age and sex using a 2017 U.S. reference population.

© 2025 Nigra AE et al. *JAMA Network Open*.

[c] Centers for Disease Control and Prevention/Agency for Toxic Substances and Disease Registry's socioeconomic vulnerability score (range 0-1, with 1 being more vulnerable). Assigned at the census tract level using birthing parent prenatal residential address.

© 2025 Nigra AE et al. *JAMA Network Open*.

eTable 2. Associations Between Prenatal Public Water Arsenic Exposure and Birth Outcomes in the ECHO Cohort (N = 13 998) With Arsenic Exposure Available (categorical, 1 μg/L higher exposure, log-doubling). Prenatal public water arsenic exposure was assigned using birthing parent residential address history during pregnancy as individual, time-weighted concentrations from ZIP Code Tabulation Area-level, population-weighted average concentrations. Models are generalized estimating equations (GEE) with participants clustered within cohort sites (n=35) using an exchangeable correlation structure. Missingness for all covariates was imputed via multiple imputation with chained equations (via the *mice* package in R; n= 5 iterations and 25 imputed datasets).

	≤0.35 μg/L	>0.35-1 μg/L	>1-2 μg/L	>2-5 μg/L	>5 μg/L[a]	Per 1 μg/L	Per log-doubling
**Birthweight (g), (n=13,882) -- mean difference (95% CI)**							
n	7,374	3,515	1,854	911	228	13,882	13,882
Model 1	0 (reference)	-28 (-76, 20)	5 (-52, 63)	22 (-40, 84)	-43 (-108, 22)	-2 (-8, 4)	0 (-16, 17)
Model 2	0 (reference)	-34 (-78, 11)	-3 (-62, 58)	17 (-46, 80)	-51 (-118, 15)	-3 (-9, 4)	-2 (-19, 16)
**Birthweight for gestational age z-score (n=13,881) – mean difference (95% CI)**							
n	7,373	3,515	1,854	911	228	13,881	13,881
Model 1	0.00 (reference)	-0.06 (-0.16 ,0.05)	0.01 (-0.07, 0.09)	0.02 (-0.11, 0.14)	-0.15 (-0.27, -0.02)	-0.00 (-0.01, 0.01)	-0.00 (-0.03, 0.03)
Model 2	0.00 (reference)	-0.07 (-0.17, 0.03)	-0.01 (-0.09, 0.08)	0.01 (-0.12, 0.14)	-0.16 (-0.29, -0.03)	-0.00 (-0.01, 0.01)	-0.01 (-0.04, 0.03)
**Gestational age (n=13,998) – percent change in geometric mean (95% CI)**							
n	7,426	3,529	1,859	948	236	13,998	13,998
Model 1	0.00 (reference)	-0.1% (-0.3, 0.2)	-0.0% (-0.5, 0.5)	0.2% (-0.2, 0.6)	0.2% (-0.3, 0.7)	0% (-0.1, 0.0)	-0.0% (-0.1 ,0.1)
Model 2	0.00 (reference)	-0.1% (-0.2, 0.2)	-0.0% (-0.6, 0.5)	0.2% (-0.2, 0.6)	0.2% (-0.3, 0.7)	0% (-0.1, 0.0)	-0.0% (-0.1, 0.1)
**Preterm birth (<37 weeks, n=13,998) – risk ratio (95% CI)**							
n	7,426	3,529	1,859	948	236	13,998	13,998
Cases	638	293	152	86	16	1,185	1,185
Model 1	1.00 (reference)	1.02 (0.85, 1.21)	1.02 (0.76, 1.36)	0.95 (0.74, 1.22)	0.76 (0.51, 1.14)	1.00 (0.98, 1.02)	0.99 (0.94, 1.06)
Model 2	1.00 (reference)	1.01 (0.86, 1.19)	1.00 (0.75, 1.33)	0.95 (0.74, 1.21)	0.75 (0.51, 1.12)	1.00 (0.98, 1.02)	0.99 (0.94, 1.05)
**Low birthweight (<2,500 g, n=13,882) – risk ratio (95% CI)**							
n	7,374	3,515	1,854	911	228	13,882	13,882
Cases	463	213	105	45	18	844	844
Model 1	1.00 (reference)	1.13 (0.83, 1.54)	1.08 (0.79, 1.47)	0.77 (0.51, 1.14)	1.38 (0.80, 2.35)	1.02 (1.00, 1.05)	1.02 (0.94, 1.11)
Model 2	1.00 (reference)	1.20 (0.88, 1.64)	1.15 (0.83, 1.58)	0.81 (0.54, 1.22)	1.46 (0.85, 2.48)	1.03 (1.01, 1.05)	1.04 (0.95, 1.13)
**Small for gestational age (n=13,881) – risk ratio (95% CI)**							
n	7,373	3,515	1,854	911	228	13,881	13,881
Cases	812	349	182	71	26	1,440	1,440
Model 1	1.00 (reference)	1.04 (0.86, 1.27)	0.99 (0.84, 1.19)	0.80 (0.62, 1.04)	1.18 (0.87, 1.59)	0.99 (0.97, 1.02)	0.98 (0.93, 1.03)
Model 2	1.00 (reference)	1.07 (0.89, 1.29)	1.04 (0.87, 1.25)	0.81 (0.62, 1.06)	1.22 (0.90, 1.65)	0.99 (0.97, 1.02)	0.98 (0.93, 1.04)

Model 1: Adjusted for birthing parent education (≤ high school degree or equivalent [ref] / some college or Associate's degree / Bachelor's degree or higher), birthing parent age (<25 [ref] / 25-29 / 30-34 / and ≥35), and infant sex.
Model 2: Further adjusted for parity, pre-pregnancy body mass index (centered), prenatal tobacco use, ZCTA population density (centered), and season of conception.
[a] Across categories, Mantel test for trend p-values were >0.05 for all models and are not reported here.

© 2025 Nigra AE et al. *JAMA Network Open*.

eTable 3. Sensitivity Analyses Evaluating the Association Between a 1 µg/L Higher Prenatal Public Water Arsenic Exposure and Birth Outcomes in the ECHO Cohort, Considering Alternative Model Adjustments and Restriction Criteria. Prenatal public water arsenic exposure was assigned using birthing parent residential address history during pregnancy as individual, time-weighted concentrations from ZIP Code Tabulation Area-level, population-weighted average concentrations. Models are generalized estimating equations (GEE) with participants clustered within cohorts (n=35) using an exchangeable correlation structure.

	**Birthweight (g)**	**Birthweight for gestational age z-score**	**Gestational age (weeks)**	**Preterm birth (<37 weeks)**	**Low birthweight (<2,500 g)**	**Small for gestational age**
	Mean difference (95% CI)	Mean difference (95% CI)	% change in geometric mean ratio (95% CI)	Risk ratio (95% CI)	Risk ratio (95% CI)	Risk ratio (95% CI)
Overall sample size	*13,882*	*13,881*	*13,998*	*13,998*	*13,882*	*13,881*
**Alternative model adjustments.** Analyses include all n=13,998 participants. Covariate missingness was imputed using multiple imputation with chained equations.						
Model 1	-2 (-8, 4)	-0.003 (-0.012, 0.007)	0% (-0.1, 0.0)	1.00 (0.98,1.02)	1.02 (1.00,1.05)	0.99 (0.97,1.02)
Model 2	-2 (-9, 4)	-0.003 (-0.014, 0.007)	0% (-0.1, 0.0)	1.00 (0.98,1.02)	1.03 (1.00,1.05)	0.99 (0.97,1.02)
Model 3	-3 (-9, 4)	-0.004 (-0.014, 0.006)	0% (-0.1, 0.0)	1.00 (0.98,1.02)	1.03 (1.01,1.05)	0.99 (0.97,1.02)
Model 4	-4 (-10, 2)	-0.006 (-0.015, 0.004)	0% (-0.1, 0.0)	1.01 (0.99,1.04)	1.03 (1.01,1.06)	1.00 (0.98,1.02)
**G-computation.** Analyses include all n=13,998 participan s. Results evaluating the effect on the outcome of increasing public drinking water arsenic exposure by one quant le						
Model 5	-3 (-24, 18)	-0.014 (-0.058, 0.029)	0.1% (-0.1, 0.2)	0.98 (0.91, 1.06)	1.01 (0.90, 1.14)	1.02 (0.94, 1.12)
**Alternative restriction criteria.** Models are adjusted for birth parent education and age, parity, pre-pregnancy body mass index (centered), prenatal tobacco use, season of birth, ZCTA population density (centered), and infant sex. (Model 2). Covariate missingness was imputed using multiple imputation with chained equations.						
**High geocoding quality**[a]	-3 (-9, 4)	-0.004 (-0.015, 0.006)	0% (-0.1, 0.0)	1.01 (0.98,1.03)	1.03 (1.01, 1.05)	0.99 (0.97, 1.02)
n	*12,910*	*12,910*	*12,910*	*12,910*	*12,910*	*12,910*
**Non-movers**[b]	-2 (-8, 4)	-0.001 (-0.011, 0.008)	0% (-0.1, 0.0)	1.01 (0.99,1.03)	1.03 (1.01, 1.05)	0.99 (0.97, 1.02)
n	*12,202*	*12,201*	*12,318*	*12,318*	*12,202*	*12,201*
**High quality exposure assessment**[c]	4 (-6 ,14)	-0.004 (-0.026, 0.018)	0.1% (0.0, 0.2)	0.97 (0.92,1.03)	0.99 (0.90, 1.09)	1.04 (0.98, 1.12)
n	*4,876*	*4,875*	*4,951*	*4,951*	*4,876*	*4,875*
**Non-movers with high quality exposure assessment**[d]	4 (-7, 14)	-0.006 (-0.03, 0.019)	0.1% (0.0, 0.2)	0.98 (0.92,1.04)	0.99 (0.90, 1.10)	1.06 (0.99, 1.14)
n	*4,123*	*4,122*	*4,198*	*4,198*	*4,123*	*4,122*
**Public water tap source**[e]	0 (-11, 11)	0.00 (-0.016, 0.016)	-0.1% (-0.2, 0.1)	0.99 (0.92,1.08)	0.98 (0.86, 1.12)	0.89 (0.71, 1.12)
n	*1,025*	*1,025*	*1,025*	*1,025*	*1,025*	*1,025*

DNC = did not converge.
Model 1: adjusted for birthing parent education and age, and infant sex.
Model 2: adjusted for birthing parent age, parity, pre-pregnancy body mass index (centered), prenatal tobacco use, season of birth, ZCTA population density (centered), and infant sex.
Model 3: adjusted for birthing parent education and age, parity, pre-pregnancy body mass index (centered), prenatal tobacco use, season of birth, ZCTA population density (centered), and infant sex.
Model 4: adjusted for birthing parent education and age, parity, pre-pregnancy body mass index (centered), prenatal tobacco use, season of birth, ZCTA population density (centered), prenatal public water uranium exposure, and infant sex.
Model 5: adjusted for birthing parent education and age, infant sex, parity, pre-pregnancy body mass index (centered), prenatal tobacco use, ZCTA population density (centered), and season of conception.
[a] Restricted to participants with the highest available geocoding quality (census tract level).
[b] Restricted to participants who did not move during pregnancy.
[c] Restricted to participants from states that publish high-quality shapefiles of public water system distribution boundaries.
[d] Restricted to participants who did not move during pregnancy who are from states that publish high-quality shapefiles of public water system distribution boundaries.
[e] Restricted to participants who reported that their home tap water source was a "public water system" or "community supply" (a subset of n=1,025/ n=1,193 with data on tap water source available). Alternative answers include "private well", "declined", and "don't know".

© 2025 Nigra AE et al. *JAMA Network Open*.

eFigure 2. Restricted Cubic Spline Models of the Association Between Prenatal Public Drinking Water Arsenic Exposure and Adverse Birth Outcomes in the ECHO Cohort (N = 13 998), With Alternative Knot Locations at the 60th and 90th Percentiles. Models are generalized estimating equations (GEE) with participants clustered within cohorts (n=35) using an exchangeable correlation structure. Model 1 (purple, solid line) is adjusted for birth parent age (categorical), and education (categorical). Model 2 (orange, dashed line) is further adjusted for parity, pre-pregnancy birth parent body mass index, prenatal tobacco use, season of conception, and ZIP Code population density. The reference is set to the 10th percentile of the public water arsenic exposure distribution. Participant prenatal water arsenic concentrations <0.35 μg/L represent non-detections; water system arsenic concentrations below the limit of detection were imputed as the limit of detection divided by the square root of two before ZCTA-level averages were generated. Although laboratories reported different limits of detection for different compliance monitoring records, the modal value of the limit of detection divided by the square root of two was 0.35 μg/L.

© 2025 Nigra AE et al. *JAMA Network Open*.

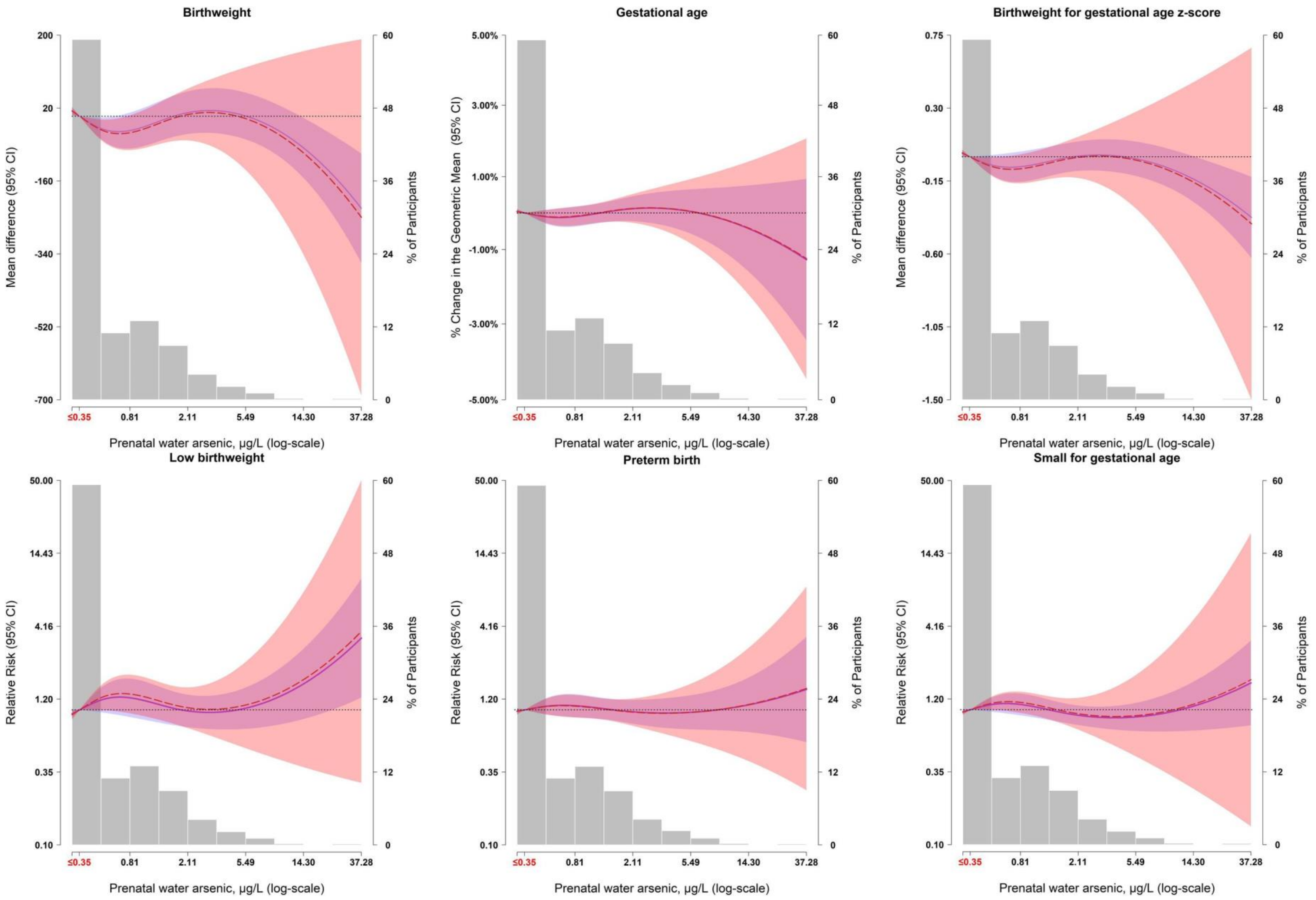

© 2025 Nigra AE et al. *JAMA Network Open*.

eFigure 3. Results of Sensitivity Analysis Removing Each Individual Cohort (Leave One Out) to Identify Influential Cohorts in the ECHO Cohort. ECHO cohort names are abbreviated and the participant sample size is provided for each cohort.

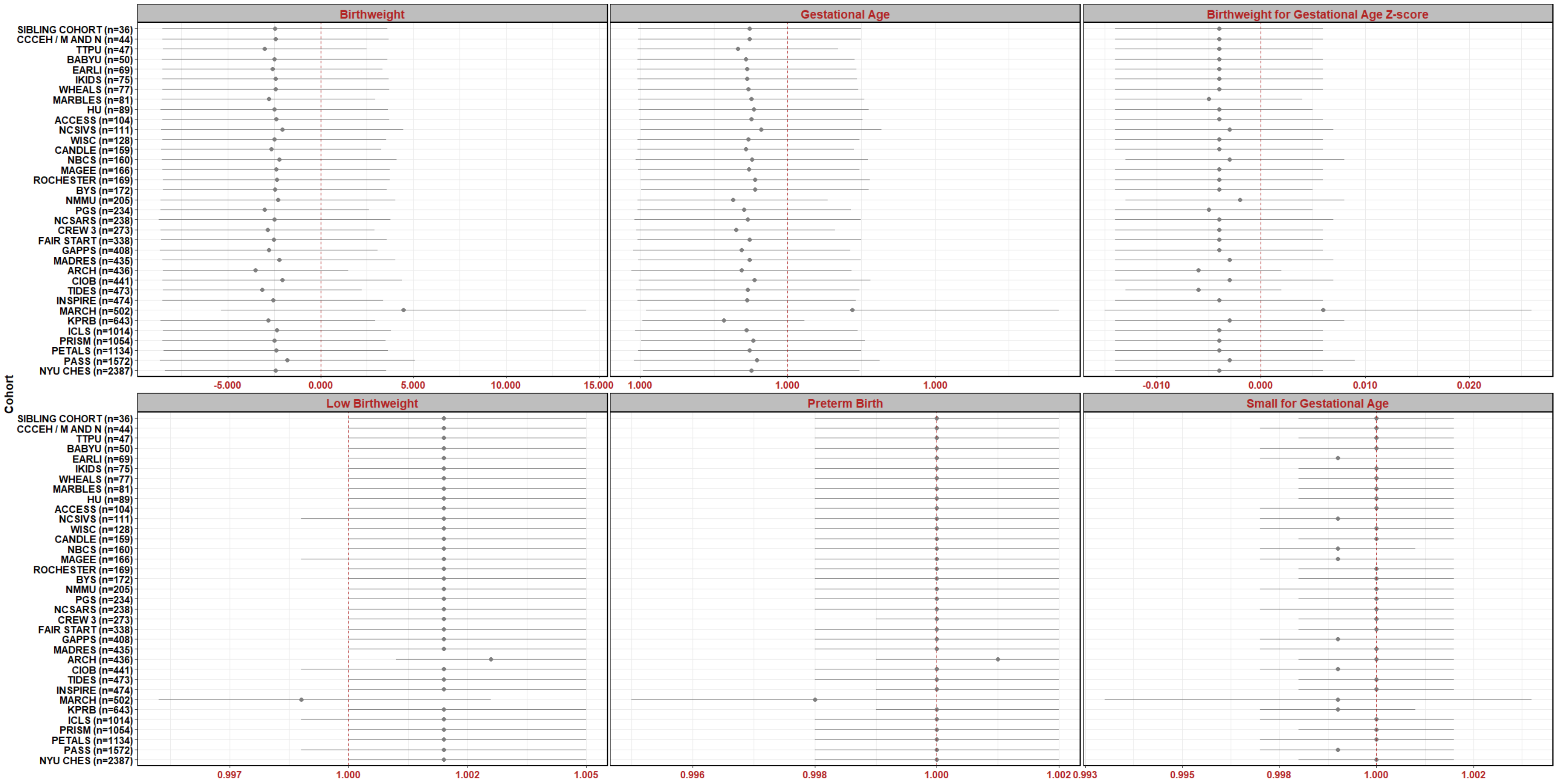

© 2025 Nigra AE et al. *JAMA Network Open*.

eFigure 4. Restricted Cubic Spline Models of the Association Between Prenatal Public Drinking Water Arsenic Exposure and Adverse Birth Outcomes in the ECHO Cohort When Evaluating Public Water Arsenic at the Tract Level. Models are generalized estimating equations (GEE) with participants clustered within cohorts (n=35) using an exchangeable correlation structure. Model 1 (purple, solid line) is adjusted for birthing parent age (categorical), and education (categorical).

Model 2 (orange, dashed line) is further adjusted for parity, pre-pregnancy birthing parent body mass index, prenatal tobacco use, season of conception, and tract population density. The reference is set to 0.35 μg/L (corresponding to undetectable public water arsenic concentrations) with knots at the 67th and 83rd percentiles of the distribution above 0.35 μg/L. Participant prenatal water arsenic concentrations <0.35 μg/L represent arsenic concentrations measured below the limit of detection, which were imputed as the limit of detection divided by the square root of two. Although laboratories reported different limits of detection for different compliance monitoring records, the modal value of the limit of detection divided by the square root of two was 0.35 μg/L.

© 2025 Nigra AE et al. *JAMA Network Open*.

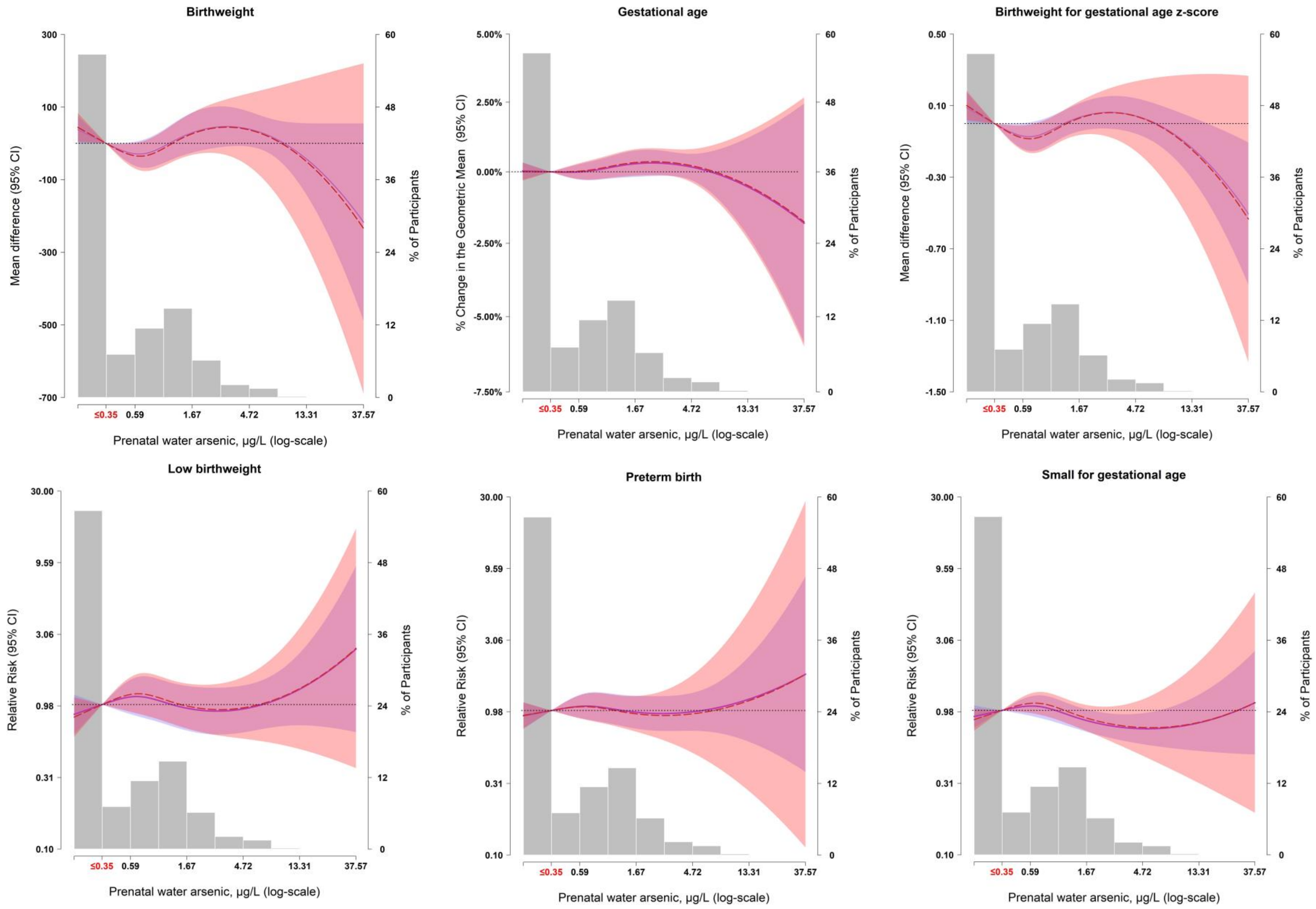

© 2025 Nigra AE et al. *JAMA Network Open*.

eTable 4. Sensitivity Analyses Evaluating the Association Between Higher Prenatal Public Water Arsenic Exposure and Birth Outcomes in the ECHO Cohort, Using Tract-Level Exposure Estimates. Arsenic exposure was modeled as across categories, per 1 µg/L higher exposure, and per log-doubling. Prenatal public water arsenic exposure was assigned using birthing parent residential address history during pregnancy as individual, time-weighted concentrations from tract-level, population-weighted average concentrations. Models are generalized estimating equations (GEE) with participants clustered within cohort sites (n=35) using an exchangeable correlation structure. Missingness for all covariates was imputed via multiple imputation with chained equations (via the *mice* package in R; n= 5 iterations and 25 imputed datasets).

	≤0.35 µg/L	>0.35-1 µg/L	>1-2 µg/L	>2-5 µg/L	>5 µg/L	Per 1 µg/L	Per log-doubling
**Birthweight (g), (n= 12,072) -- mean difference (95% CI)**							
n	6,769	2,816	1,503	786	198	12,072	12,072
Model 1	0 (reference)	-8 (-56, 40)	26 (-41, 94)	65 (-17,147)	-33 (-110, 43)	3 (-4, 10)	10 (-4, 24)
Model 2	0 (reference)	-14 (-61, 34)	20 (-50, 91)	66 (-7, 137)	-38 (-116, 40)	3 (-5, 11)	9 (-6, 23)
**Birthweight for gestational age z-score (n=12,071) – mean difference (95% CI)**							
n	6,768	2,816	1,503	786	198	12,071	12,071
Model 1	0.00 (reference)	-0.032 (-0.124, 0.061)	0.031 (-0.069, 0.131)	0.117 (0.007, 0.228)	-0.159 (-0.299, -0.018)	0.004 (-0.008, 0.016)	0.011 (-0.016, 0.037)
Model 2	0.00 (reference)	-0.043 (-0.127, 0.041)	0.016 (-0.090, 0.122)	0.122 (0.021, 0.223)	-0.168 (-0.307, -0.030)	0.004 (-0.009, 0.017)	0.009 (-0.020, 0.038)
**Gestational age (n=12,162) – percent change in geometric mean (95% CI)**							
n	6,809	2,826	1,507	816	204	12,162	12,162
Model 1	0.00 (reference)	0.1% (-0.2, 0.4)	0.3% (-0.3, 0.9)	0.2% (-0.4, 0.8)	0.2% (-0.5, 0.9)	0.0% (-0.1, 0.1)	0.1% (-0.1, 0.2)
Model 2	0.00 (reference)	0.1% (-0.2, 0.5)	0.3% (-0.2, 0.9)	0.3% (-0.3, 0.8)	0.3% (-0.4, 0.9)	0.0% (-0.1, 0.1)	0.1% (0.0, 0.2)
**Preterm birth (<37 weeks, n=12,162) – risk ratio (95% CI)**							
n	6,809	2,826	1,507	816	204	12,162	12,162
Cases	587	242	125	80	16	1,050	1,050
Model 1	1.00 (reference)	1.07 (0.90, 1.30)	0.99 (0.69, 1.40)	1.05 (0.74, 1.50)	0.89 (0.59, 1.35)	1.01 (0.98, 1.04)	1.00 (0.94, 1.07)
Model 2	1.00 (reference)	1.06 (0.87, 1.28)	0.96 (0.68, 1.35)	1.00 (0.73, 1.38)	0.86 (0.57, 1.31)	1.00 (0.97, 1.04)	0.99 (0.93, 1.05)
**Low birthweight (<2,500 g, n=12,072) – risk ratio (95% CI)**							
n	6,769	2,816	1,503	786	198	12,072	12,072
Cases	438	163	87	41	14	743	743
Model 1	1.00 (reference)	1.04 (0.79, 1.37)	1.02 (0.66, 1.57)	0.81 (0.42, 1.57)	1.16 (0.69, 1.97)	1.01 (0.96, 1.06)	0.99 (0.89, 1.10)
Model 2	1.00 (reference)	1.08 (0.81, 1.44)	1.06 (0.69, 1.63)	0.81 (0.43, 1.54)	1.18 (0.71, 1.95)	1.01 (0.97, 1.06)	1.00 (0.90, 1.10)
**Small for gestational age (n=12,071) – risk ratio (95% CI)**							
n	6,768	2,816	1,503	786	198	12,071	12,071
Cases	763	274	136	62	20	1,255	1,255
Model 1	1.00 (reference)	0.96 (0.83, 1.12)	0.83 (0.66, 1.04)	0.77 (0.58, 1.02)	0.95 (0.65, 1.39)	0.95 (0.90, 1.00)	0.93 (0.88, 1.00)
Model 2	1.00 (reference)	1.01 (0.88, 1.16)	0.87 (0.70, 1.08)	0.78 (0.58, 1.05)	0.98 (0.67, 1.45)	0.95 (0.90, 1.01)	0.95 (0.88, 1.01)

Model 1: Adjusted for birthing parent education (≤ high school degree or equivalent [ref] / some college or Associate's degree / Bachelor's degree or higher), birthing parent age (<25 [ref] / 25-29 / 30-34 / and ≥35), and infant sex.
Model 2: Further adjusted for parity, pre-pregnancy body mass index (centered), prenatal tobacco use, tract population density (centered), and season of conception.

© 2025 Nigra AE et al. *JAMA Network Open*.

eFigure 5. Sensitivity Analyses Evaluating the Association Between Higher Prenatal Public Water Arsenic Exposure and Birth Outcomes in the ECHO Cohort, Using Tract-Level Exposure Estimates. Associations represent effect estimates per a 1 µg/L higher prenatal public water arsenic exposure in linear models. The size of the square corresponds to the number of participants in each subgroup. Prenatal public water arsenic exposure was assigned using birthing parent residential address history during pregnancy as individual, time-weighted concentrations from tract-level, population-weighted average concentrations. Models are generalized estimating equations (GEE) with participants clustered within cohorts (n=34) using an exchangeable correlation structure. Missingness for all covariates was imputed via multiple imputation with chained equations (via the mice package in R; n=5 iterations and 10 imputed datasets). Models are adjusted for birthing parent education, age, parity, pre-pregnancy birthing parent body mass index, prenatal tobacco use, season of birth, and tract population density. Effect estimates are not presented for subgroup with less than 50 cases or when models did not converge. AI/AN/NH/PI = American Indian, Alaskan Native, Native Hawaiian, Pacific Islander; Birthing parent socioeconomic vulnerability was assessed using the CDC/ATSDR's census tract-level socioeconomic vulnerability index. *We do not report results for subgroups with less than 50 events or when models did not converge because of small sample size.

© 2025 Nigra AE et al. *JAMA Network Open*.

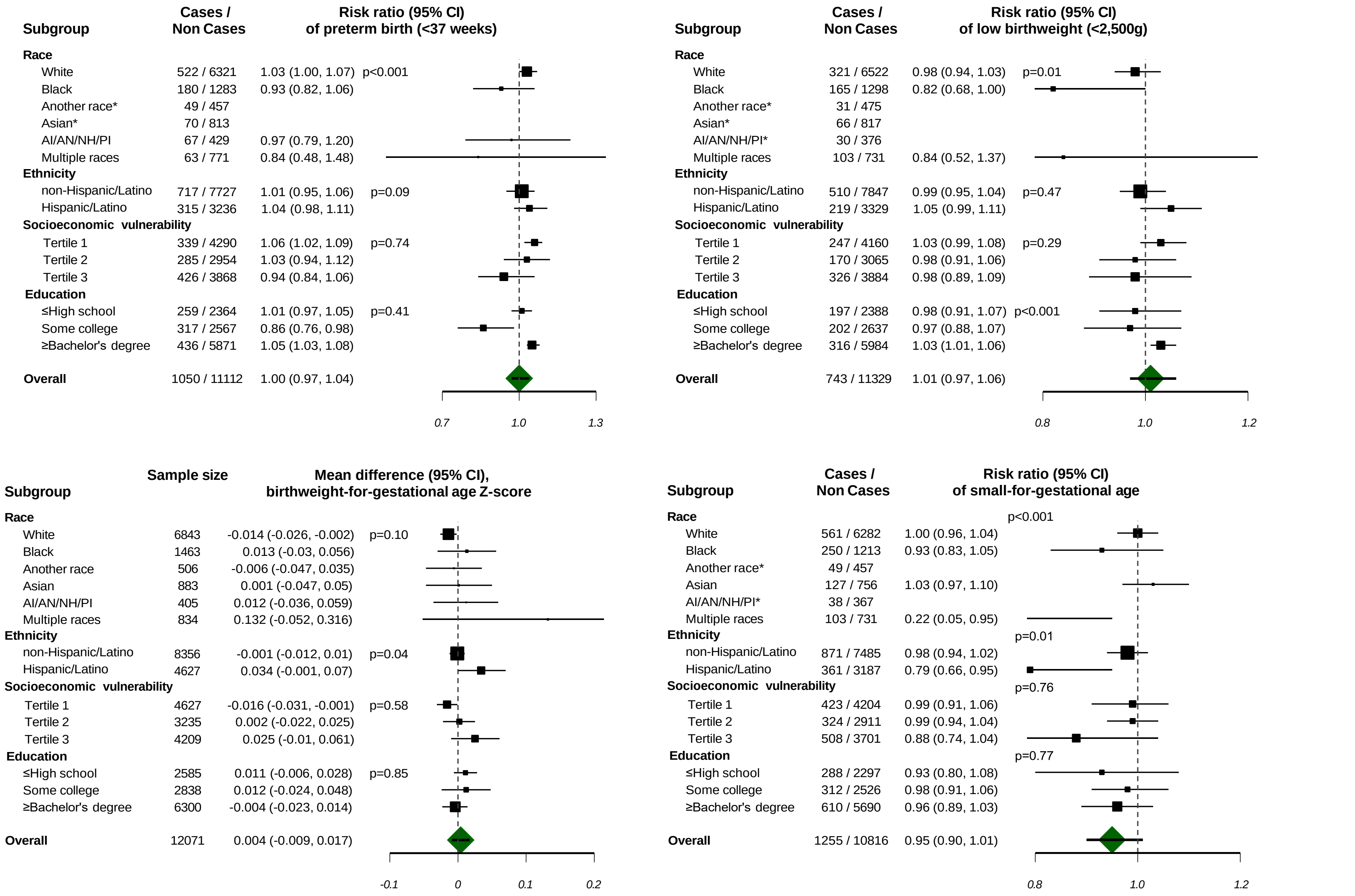

© 2025 Nigra AE et al. *JAMA Network Open*.

eFigure 6. Directed Acyclic Graph illustrating (A) the proposed association between public water arsenic and birth outcomes, and (B) potential mediation of racial/ethnic disparities in birth outcomes by public drinking water arsenic. In (A), we consider parent educational attainment and race and ethnicity as potential effect measure modifiers.

(A)

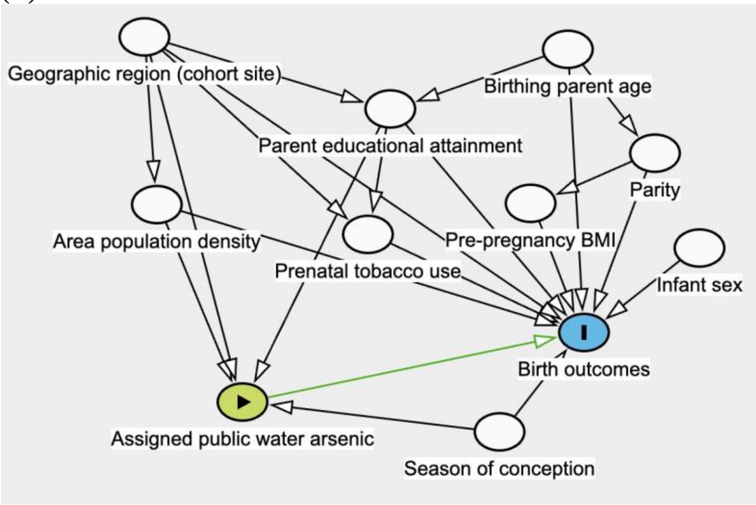

(B)

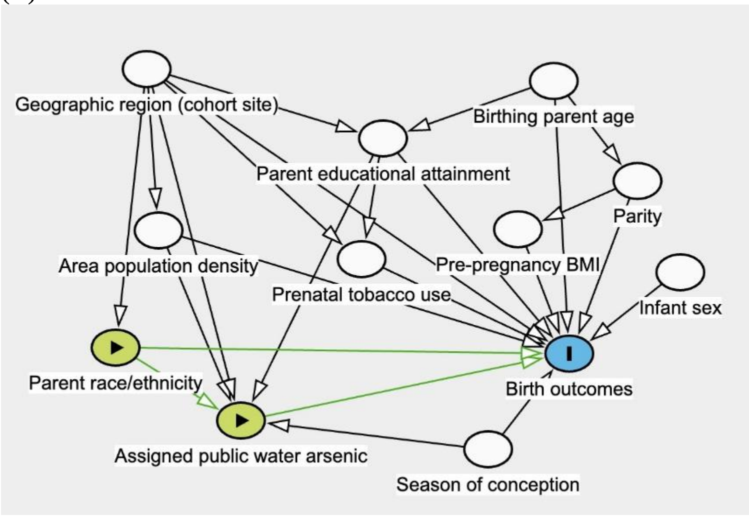

© 2025 Nigra AE et al. *JAMA Network Open*.

eReferences

1. Bloomquist TR, Spaur M, Cerna-Turoff I, et al. Public drinking water contaminant estimates for birth cohorts in the Environmental Influences on Child Health Outcomes (ECHO) Cohorts. *Journal of Exposure Sciences and Environmental Epidemiology*. 2024:1-13.
2. Spaur M, Glabonjat RA, Schiling K, et al. Contribution of arsenic and uranium in private wells and community water systems with urinary biomarkers in US adults: The Multi-Ethnic Study of Atherosclerosis and the Strong Heart Family Study. *Journal of Exposure Sciences and Environmental Epidemiology*. 2023;34(1):77-89.
3. Nigra AE, Chen, Q., Chillrud, S. N., Wang, L., Harvey, D., Mailloux, B., Factor-Litvak, P., & Navas-Acien, A. Inequalities in public water arsenic concentrations in counties and community water systems across the United States, 2006-2011. *Environmental Health Perspectives*. 2020;doi:https://doi.org/10.1289/EHP7313.
4. U.S. Environmental Protection Agency. The analysis of regulated contaminant occurrence data from public water systems in support of the Third Six-Year Review of National Primary Drinking Water Regulations: Chemical Phase Rules and Radionuclides Rules. Accessed 20 July 2020, Available: https://www.epa.gov/sites/production/files/2016-12/documents/810r16014.pdf
5. U.S. Environmental Protection Agency. Six-Year Review 3 Compliance Monitoring Data (2006-2011). Accessed 20 July 2020, https://www.epa.gov/dwsixyearreview/six-year-review-3-compliance-monitoring-data-2006-2011
6. United States Environmental Protection Agency. The data management and quality assurance/quality control process for the Third Six-Year Review Information Collection Rule Dataset. Accessed 20 July 2020, Available: https://www.epa.gov/sites/default/files/2016-12/documents/810r16015_0.pdf
7. U.S. Environmental Protection Agency. The Standardized Monitoring Framework: A quick reference guide. Accessed 20 July 2020, https://nepis.epa.gov/Exe/ZyPDF.cgi/3000667K.PDF?Dockey=3000667K.PDF
8. Nigra AE, Sanchez TR, Nachman KE, et al. The effect of the Environmental Protection Agency maximum contaminant level on arsenic exposure in the USA from 2003 to 2014: An analysis of the National Health and Nutrition Examination Survey (NHANES). *The Lancet Public Health*. 2017;2(11)(11):e513-e521. doi:10.1016/s2468-2667(17)30195-0
9. Spaur M, Bostick BC, Chillrud SN, Factor-Litvak P, Navas-Acien A, Nigra AE. Impact of lowering the US maximum contaminant level on arsenic exposure: Differences by race, region, and water arsenic in NHANES 2003-2014. *Environmental Pollution*. 2023;333:122047. doi:10.1016/j.envpol.2023.122047
10. McDonald YJ, Anderson KM, Caballero MD, et al. A systematic review of geospatial representation of United States community water systems. *AWWA Water Science*. 2022;4(1):e1266.
11. United States Environmental Protection Agency. Community Water System Service Area Boundaries. Accessed 11 September 2024, Available: https://www.epa.gov/ground-water-and-drinking-water/community-water-system-service-area-boundaries
12. Ravalli F, Yuanzhi Y, Bostick BC, et al. Sociodemographic inequalities in uranium and other metals in community water systems across the US, 2006-2011. *Lancet Planetary Health*. 2022;6(4):e320-e330.

© 2025 Nigra AE et al. *JAMA Network Open*.

13. Beene D, Collender P, Cardenas A, et al. A mass-balance approach to evaluate arsenic intake and excretion in different populations. *Environment International*. 2022;166:107371.

14. United States Environmental Protection Agency. National Primary Drinking Water Regulations: Arsenic and clarifications to compliance and new source contaminants monitoring. Federal Register: 66 Fed. Reg. 6976. National Archives and Records Administration. https://www.govinfo.gov/content/pkg/FR-2001-01-22/pdf/FR-2001-01-22.pdf

15. United States Environmental Protection Agency. The data management and quality assurance/quality control process for the Fourth Six-Year Review Information Collection Request Dataset. Accessed 20 August 2024, Available: https://www.epa.gov/system/files/documents/2024-03/syr4-data-management-and-quality-assurance_508.pdf

16. Centers for Disease Control and Prevention/ Agency for Toxic Substances and Disease Registry/ Geospatial Research Analysis and Services Program. CDC/ATSDR Social Vulnerability Index 2018 Database US. Accessed 16 December 2021, https://www.atsdr.cdc.gov/placeandhealth/svi/data_documentation_download.html

17. Zou G. A modified poisson regression approach to prospective studies with binary data. *American Journal of Epidemiology*. 2004;159(7):702-706.

18. Martenies SE, Zhang M, Corrigan AE, et al. Developing a National-Scale Exposure Index for Combined Environmental Hazards and Social Stressors and Applications to the Environmental Influences on Child Health Outcomes (ECHO) Cohort. *International Journal of Environmental Research and Public Health*. 2023;20(14):6339.

19. Swilley-Martinez ME, Coles SA, Miller VE, et al. “We adjusted for race”: now what? A systematic review of utilization and reporting of race in American Journal of Epidemiology and Epidemiology, 2020–2021. *Epidemiologic Reviews*. 2023;45(1):15-31.
