# Supplementary material for: Public Water Arsenic and Birth Outcomes in the Environmental Influences on Child Health Outcomes Cohort
Source: JAMA Netw Open. 2025 Jun 16;8(6):e2514084. doi: 10.1001/jamanetworkopen.2025.14084 (PMC12171937; doi:10.1001/jamanetworkopen.2025.14084)
Supplement: Supplement 3. — Data Sharing Statement [file jamanetwopen-e2514084-s003.pdf]

Data Sharing Statement

Nigra. Public Water Arsenic and Birth Outcomes in the Environmental Influences on Child Health Outcomes Cohort. *JAMA Netw Open*. Published June 09, 2025. doi:10.1001/jamanetworkopen.2025.14084

Data
Data available: Yes
Data types: Deidentified participant data
How to access data: Select de-identified data from the ECHO Program are available through NICHD's Data and Specimen Hub (DASH). Information on study data not available on DASH, such as some Indigenous datasets, can be found on the ECHO study DASH webpage.
When available: beginning date: 11-01-2024

Supporting Documents
Document types: None

Additional Information
Who can access the data: Select de-identified data from the ECHO Program are available through NICHD's Data and Specimen Hub (DASH). Information on study data not available on DASH, such as some Indigenous datasets, can be found on the ECHO study DASH webpage.
Types of analyses: Any purposes.
Mechanisms of data availability: Data on the DASH platform are publicly available.
Any additional restrictions: None.
